# Nanobody Nb07 mitigates sepsis by blocking the PFKM-p53-PD-1 axis to enhance macrophage phagocytosis

**DOI:** 10.7150/thno.124303

**Published:** 2026-01-01

**Authors:** Binbin Ji, Hui Guo, Rong Xing, Miaomiao Sun, Yu Cheng, Chen Yao, Hanyong Zhu, Xuerong Wang, Ruihan Jiang, Xin Chen, Zimeng Liu, Suyan Wang, Fei Xu, Fangyu Zhang, Fuxing Dong, Xiucheng Pan, Jing Yang, Yuchen Pan

**Affiliations:** 1 Jiangsu International Laboratory of Immunity and Metabolism, Jiangsu Province Key Laboratory of Immunity and Metabolism, Department of Pathogenic Biology and Immunology, Xuzhou Medical University, Xuzhou 221004, Jiangsu, China.; 2 National Demonstration Center for Experimental Basic Medical Science Education, Xuzhou Medical University, Xuzhou 221004, Jiangsu, China.; 3 Department of Clinical Laboratory, Affiliated Jinhua Hospital, Zhejiang University School of Medicine, Jinhua 310058, Zhejiang, China.; 4 Public Experimental Research Center, Xuzhou Medical University, Xuzhou 221004, Jiangsu, China.; 5 Department of Infectious Disease, The Affiliated Hospital of Xuzhou Medical University, Xuzhou Medical University, Xuzhou 221004, Jiangsu, China.

**Keywords:** sepsis, PFKM, moonlighting function, macrophage, phagocytosis

## Abstract

**Rationale:** Macrophage phagocytosis is essential for pathogen clearance during sepsis. We previously demonstrated that the glycolytic enzyme 6-phosphofructokinase, muscle type (PFKM), modulates macrophage functions and its deficiency alleviates sepsis in mice. However, the function of PFKM in regulating macrophage phagocytosis remains unclear.

**Methods:** CD14^+^ monocytes were sorted by flow cytometry from healthy volunteers and septic patients, and the subcellular localization of PFKM was assessed by immunofluorescence. Nuclear translocation mechanisms and PFKM-p53 interaction were identified by Co-immunoprecipitation coupled with mass spectrometry (Co-IP/MS) and validated by Co-IP. Transcriptomic sequencing was used to identify the downstream target of the PFKM-p53 complex. Inflammatory cytokine levels were detected by ELISA and real-time RT-PCR, and the phagocytosis of macrophages was assessed by flow cytometry. Dual-luciferase reporter assays and ChIP were employed to investigate whether PFKM acts as a co-regulator of p53 in mediating *Pdcd1* transcription. Nanobodies targeting PFKM-p53 were screened and subsequently synthesized according to the sequences. The effect of nuclear PFKM and the therapeutic effect of nanobodies were evaluated on the well-established sepsis mouse models induced by *Escherichia coli* or cecal ligation and puncture.

**Results:** PFKM translocated to the macrophage nucleus during sepsis. Nuclear accumulation of PFKM impaired phagocytosis through a non-glycolytic “moonlighting” function and exacerbated sepsis. Mechanistically, PFKM interacts with p53, which facilitates its nuclear translocation. Subsequently, PFKM promotes p53 acetylation at K120, enhancing p53 binding to the *Pdcd1* promoter and driving its transcription, thereby suppressing macrophage phagocytosis. Blocking the PFKM-p53 interaction with a nanobody, Nb07, restored phagocytosis of macrophages and alleviated sepsis in mice.

**Conclusion:** Our data reveal the PFKM-p53-PD-1 axis that suppresses macrophage phagocytosis in sepsis and highlight the therapeutic potential of targeting this pathway with nanobody-based strategies.

## Introduction

Sepsis is one of the adverse clinical outcomes characterized as life-threatening organ dysfunction 1, posing a major global socioeconomic burden 2,3. During sepsis, macrophage phagocytosis is critical for pathogen clearance, tissue regeneration, and inflammation resolution 4. Both macrophage deletion and phagocytosis dysfunction could exacerbate sepsis 5,6, while enhancing macrophage phagocytosis improves the survival and alleviates multi-organ injury in murine models 7,8. However, infection can induce macrophage phagocytic dysfunction 9,10, and its underlying mechanisms remain unclear.

Macrophage functions, including phagocytosis, are closely related to glycolysis 11. Emerging evidence indicates that properly modulating macrophage glycolysis could represent a promising strategy for sepsis 12,13. Phosphofructokinase 1 (PFK1), the key rate-limiting enzyme modulating glycolysis 14, exists as cell-specific isoforms. We previously reported that the protein abundance of the muscle-type isoenzyme (PFKM) was higher than that of liver type (PFKL) and platelet type (PFKP) 15. The PFKM protein level was elevated in mono-macrophages from septic patients, and PFKM knockout conferred protection against sepsis in mice 15. Notably, although PFKM is conventionally regarded as a cytoplasmic protein (https://www.proteinatlas.org), we observed PFKM nuclear translocation in stimulated macrophages. Given the critical influence of subcellular localization on protein functions, the role of nuclear PFKM is indistinct.

While current research on PFKM primarily focuses on its canonical metabolic function, glycolytic enzymes exhibit non-canonical “moonlighting” functions 16. For example, Pyruvate kinase M2 (PKM2) translocates to the nucleus 17, where it interacts with the transcription factors to enhance the expression of target genes 18. Recent research has shown that PFKM stabilizes the target protein in glioblastoma cells 19. Nevertheless, the potential for nuclear PFKM to exert moonlighting functions, specifically in macrophage phagocytosis, remains unexplored.

In this study, we investigated the role and underlying mechanisms of nuclear PFKM in regulating macrophage phagocytosis. Our data indicated that nuclear PFKM accumulation impaired macrophage phagocytosis by interacting with p53 and enhancing p53-mediated PD-1 expression. Furthermore, we developed a nanobody, Nb07, to block this specific interaction. Nb07 treatment restored macrophage phagocytosis and alleviated sepsis in mice, which provides a potential therapeutic strategy for sepsis.

## Materials and Methods

### Reagents

Lipopolysaccharides (LPS) (O127:B8, Sigma), macrophage colony-stimulating factor (M-CSF) (APA090Mu61, Cloud-Clone Corp), 5-fluorouracil (5-FU) (F0101-5G, BOSF), and anti-PD-1 monoclonal antibody (BE0273, Bio X Cell) were prepared in PBS (BC-BPBS-01, Bio-Channel). C646 (p300/CBP inhibitor, HY-13823, MCE), DOX (HY-N0565, MCE), fluorescein isothiocyanate (FITC) (F104848-1g, Aladdin), Ivermectin (IVM) (importin inhibitor, HY-15310, MCE), and MG149 (Tip60 inhibitor, HY-15887, MCE) were prepared in DMSO.

### Patient samples

Peripheral blood samples (n = 5) were obtained as described 15 with the approval of the Ethics Committee of Xuzhou Medical University (XYFY2022-KL442-01).

### Mice

C57BL/6 mice were purchased from GemPharmatech Co, Ltd (N000013). Homozygote *Pfkm^f/f^; Lyz^Cre/+^* mice were obtained as described 15. The p53 knockout mice were obtained from Jackson Laboratory (JAX: 002101).

All mice were maintained under SPF conditions with a 12 h light/dark cycle and provided with a standard diet (1002, Pizhou Xiaohe Technology Development Co, Ltd) and water ad libitum, with the approval of the Institutional Animal Care and Use Committee of Xuzhou Medical University (202303T015).

### Sepsis models

For the *Escherichia coli* (*E. coli*)-induced sepsis model, C57BL/6, *Pfkm^f/+^* or *Pfkm^f/f^; Lyz^Cre/+^* mice (8-10 weeks old) were injected intraperitoneally with 3-5 × 10⁷ CFU clinical *E. coli* 7.

LPS-induced sepsis model and cecal ligation and puncture (CLP)-induced sepsis model were performed as previously described 15.

To overexpress nuclear PFKM in macrophages *in vivo*, the *Pfkm^f/+^* or *Pfkm^f/f^; Lyz^Cre/+^* mice (8-10 weeks old) were intravenously injected with adeno-associated virus (AAV9-NLS-PFKM-3×Flag, Zebrafish Biotechnology Co, Ltd.) at a dose of 1 × 10^11^ VG/mouse.

For Nb07 treatment, mice were injected with 1 mg/kg Nb07 intraperitoneally 2 h before or 6 h after modeling. Mice intraperitoneally injected with PBS (BC-BPBS-01, Bio-Channel) served as the control group. Mortality was monitored and recorded daily following the injection.

### Cells

Peripheral blood mononuclear cells (PBMNCs) were isolated with Percoll (17-0891-02, GE HealthCare) following the manufacturer's instructions. HEK-293T cells (FH0244) and RAW264.7 (FH0328) were obtained from FuHeng Cell Center and cultured in DMEM (KGL1206-500, Keygen) with 10% FBS (#3022A, Umedium, Hefei, China).

Bone marrow cells were harvested from the femurs and tibias of mice. The bone marrow cells were cultured in DMEM with 10% FBS and 20 ng/mL M-CSF (APA090Mu61, Cloud-Clone Corp) for 4 days to obtain bone marrow-derived macrophages (BMDMs). The abdominal cavity was lavaged with PBS to obtain peritoneal macrophages. All cells were maintained at 37 °C with 5% CO₂.

### Western blotting

Western blotting was carried out as described 15. Briefly, the cells were lysed using RIPA lysis buffer (P0013C, Beyotime) and then separated by 8% SDS-PAGE. The proteins were transferred onto a nitrocellulose membrane (B500, Abm). Antibodies are listed in **Table S1**. Images were obtained using an ECL system (BIO-RAD) and analyzed by Image Lab.

### RNA extraction and real-time RT-PCR

Total RNA was extracted using Trizol Reagent (15596026, Invitrogen™) and reverse-transcribed to cDNA with 5 × All-In-One RT MasterMix (G490, Abm). Real-time RT-PCR was performed as described 15. Gene expression was calculated using the 2^-△△Ct^ method with *Actb* as the internal control. Primer sequences are listed in **Table S2**.

### Transcriptomic sequencing

RAW264.7 overexpressing nuclear PFKM were treated with or without 600 ng/mL DOX for 48 h. Then, the total RNA from these two groups (n = 3) was extracted as described above. The RNA samples were submitted to Majorbio (Shanghai, China) for mRNA enrichment, purification, and subsequent transcriptomic sequencing. The data were analyzed by the Majorbio platform (www.majorbio.com/tools).

### Multiple immunofluorescence staining

Fixed cells and tissue sections were stained with the indicated antibodies (**Table S1**) using the PANO6-plex IHC kit (TSA-Rab, 0081100100, Panovue) according to the manufacturer's protocols. Fluorescent images were captured using a laser scanning confocal microscope (Leica STELLARIS 5, Germany).

The nuclear translocation ratio was quantified for each sample by analyzing three randomly selected fields of view. The nuclear translocation ratio was calculated as the proportion of CD14^+^ or F4/80^+^ cells exhibiting nuclear PFKM localization among the total number of respective cells per field. The results from five samples per group are presented as the mean of the three field measurements.

### Histology analysis

Lung tissues were fixed in 4% PFA (VIH100, VICMED) for over 24 h, followed by dehydration, embedding, sectioning, and hematoxylin and eosin (H & E) staining for histological examination. Score the alveolar wall thickness, lung tissue damage, and inflammatory cell infiltration on a 5-point scale (0 = minimal injury, 1 = mild injury, 2 = moderate injury, 3 = severe injury, 4 = maximum injury). The sum of these scores determines the severity of lung injury.

### Bacterial load determination

The peritoneal cavity was flushed with 5 mL PBS to obtain peritoneal lavage fluid. Peripheral blood was collected from the orbital venous plexus and diluted 1:50 in PBS. All samples were plated onto LB agar plates (VIC454, VICMED) and incubated at 37 °C for 18 h.

### Biochemical analysis

IL-6 (1210602, Dakewe) and TNF-α (430904, BioLegend) levels were measured using ELISA according to the manufacturer's protocols.

### Lactate measurement

Cell supernatants were collected when cells reached 70-80% confluence. Lactate levels were measured using the Lactate Assay Kit (KGA7402, KeyGEN). The lactate concentration was calculated with the formula: (sample-blank)/(standard-blank) × standard concentration × dilution factor.

### Cytometric bead array (CBA)

Cytokine concentrations were measured using the LEGENDplex™ CBA kit (740446, BioLegend) according to the manufacturer's protocols. In brief, the samples were incubated with beads and determined by flow cytometry. Data were analyzed using LEGENDplex™ data analysis software (v8.0).

### Co-immunoprecipitation

Co-IP was performed as described previously 20. Briefly, cells were lysed using RIPA lysis buffer, then incubated with the appropriate antibodies overnight, followed by incubation with Protein A/G agarose beads (20334 & 20399, Thermo Fisher Scientific) for another 2 h at 4 °C. The beads were washed, and the proteins were eluted by boiling in 1 × SDS-PAGE protein loading buffer (20315ES, YEASEN). The eluted proteins were determined by Western blotting. Detailed antibody information is provided in **Table S1**.

### Nuclear and cytoplasmic separation

Cells were fractionated into cytoplasmic and nuclear components using the Proteintech kit (PK10014, Proteintech) according to the manufacturer's protocols. After centrifugation, the supernatant containing cytoplasmic proteins and the pellet containing nuclear proteins were harvested for Western blotting.

### Dual-luciferase reporter assay

For dual-luciferase reporter assays, HEK-293T cells were co-transfected with a *Pdcd1* promoter-driven firefly luciferase plasmid, the Renilla luciferase control plasmid-pRL-TK (D2690, Beyotime), and expression vectors or empty controls using jetPRIME^®^ transfection reagent (0000004162, Polyplus). Firefly luciferase activity was measured with the Bio-Lumi^TM^ kit (RG029s, Beyotime) and normalized to Renilla luciferase activity to account for transfection efficiency.

### Flow cytometry

Cells were harvested and washed twice with cold PBS. Then, cells were incubated with fluorochrome-conjugated antibodies for 30 min at 4 °C (**Table S1**). Afterwards, cells were detected by BD FACSCanto^TM^ II flow cytometer and analyzed using FlowJo software.

### Screening and synthesis of nanobodies

First, the interacting sites between PFKM and p53 were predicted through AlphaFold3-based structural modeling. Subsequently, 300 novel peptide binders were designed de novo to mimic CDR3 regions using RFdiffusion. Then, binders were screened via HDock molecular docking, grafted onto a nanobody scaffold, and generated variant nanobodies by computational mutagenesis. High-affinity nanobodies targeting the PFKM protein were screened by Hefei Kejing Biotechnology Co., Ltd. Finally, through one-to-one comparison by AlphaFold3, the nanobodies (Nb07, Nb15, Nb51) that could completely occupy most of the PFKM-p53 binding sites were synthesized by Biointron Biotechnology Inc.

### Synthesis of Nb07-FITC

First, Nb07 (10 mg) and FITC (1 mg, F104848, Aladdin) were added to a tube containing 2 mL PBS. The mixture was stirred overnight at 4 °C and then dialyzed against 4 L of ddH_2_O in a 500 Da MWCO (MD34-500, VICMED) for two days to remove the unreacted FITC. The products were dried and dissolved in PBS to obtain the fluorescent nanobody.

### Cell viability

Cells were seeded into 96-well plates at 10^4^ cells/well and treated with different concentrations of Nb07. After 24 h, 10 µL Cell Counting Kit-8 (C6005, NCM) was added to each well and incubated for 30 min. The absorbance values at 450 nm were detected by the Synergy2 Multimode Microplate Reader.

### Bacterial phagocytosis assay

The phagocytosis assay was performed by pHrodo™ iFL Red or Green STP Ester (P36010 and P36012, Thermo Fisher) according to instructions as described previously 21. Briefly, *E. coli* were labeled with pHrodo and then co-cultured with macrophages at an MOI of 20:1 for 1 h at 37 °C. Subsequently, cells were washed 3 times with PBS, and phagocytosis was analyzed by flow cytometry (BD FACSCanto™ II). Data were analyzed using FlowJo software.

### Chromatin immunoprecipitation (ChIP) assay

The binding sites of p53 on the *Pdcd1* promoter were predicted by Transcription Factor Affinity Prediction (TRAP 3.05). ChIP was performed using the BeyoChIP™ ChIP assay kit with Protein A/G Magnetic Beads (P2080S, Beyotime) according to the manufacturer's instructions as described 22. Immunoprecipitation of cross-linked chromatin was conducted with antibodies (**Table S1**). The purified DNA was amplified by real-time PCR using primers listed in **Table S3**.

### Plasmid, siRNAs, lentivirus, and transfection

The nuclear localization signal (NLS)-PFKM and PD-1 overexpression plasmids were constructed by cloning the respective cDNAs into the pLVX-TetOne-Puro vector, which was kindly provided by Professor Feng Guo (Xuzhou Medical University). Subsequently, lentiviruses were constructed, and stable cell lines were established following standard protocols 23. Additionally, PFKM, NLS-PFKM, p53, or Nb07 cDNAs were amplified and cloned into the pcDNA3.1 vector. The shRNA plasmids targeting p53 (shp53#1 and shp53#2) were gifts from Professor Jiehui Di (Xuzhou Medical University). The siRNAs against PD-1 and p53 were purchased from HyCyte^TM^ (Suzhou, China). The adeno-associated virus (AAV) for nuclear PFKM overexpression in mouse macrophages was procured from Zebrafish Biotech (Nanjing, China).

### Statistical analysis

Data are presented as Mean ± SD. Survival curve data are presented as a Kaplan-Meier plot, with a log-rank test used to compare susceptibility between the different groups. One-way ANOVA was applied for multi-group comparisons, and t-tests for two-group comparisons. Two-way ANOVA was applied for two-factor comparisons. Experiments were performed at least twice. The number of mice or samples per group (replicates of independent experiments) and statistical tests are shown in the Figure legends. Statistical differences were defined as *P < 0.05, **P < 0.01, and ***P < 0.001, analyzed by GraphPad Prism 8.0 (GraphPad Software Inc.).

## Results

### Nuclear accumulation of PFKM in macrophages exacerbates sepsis

**Figure 1:**
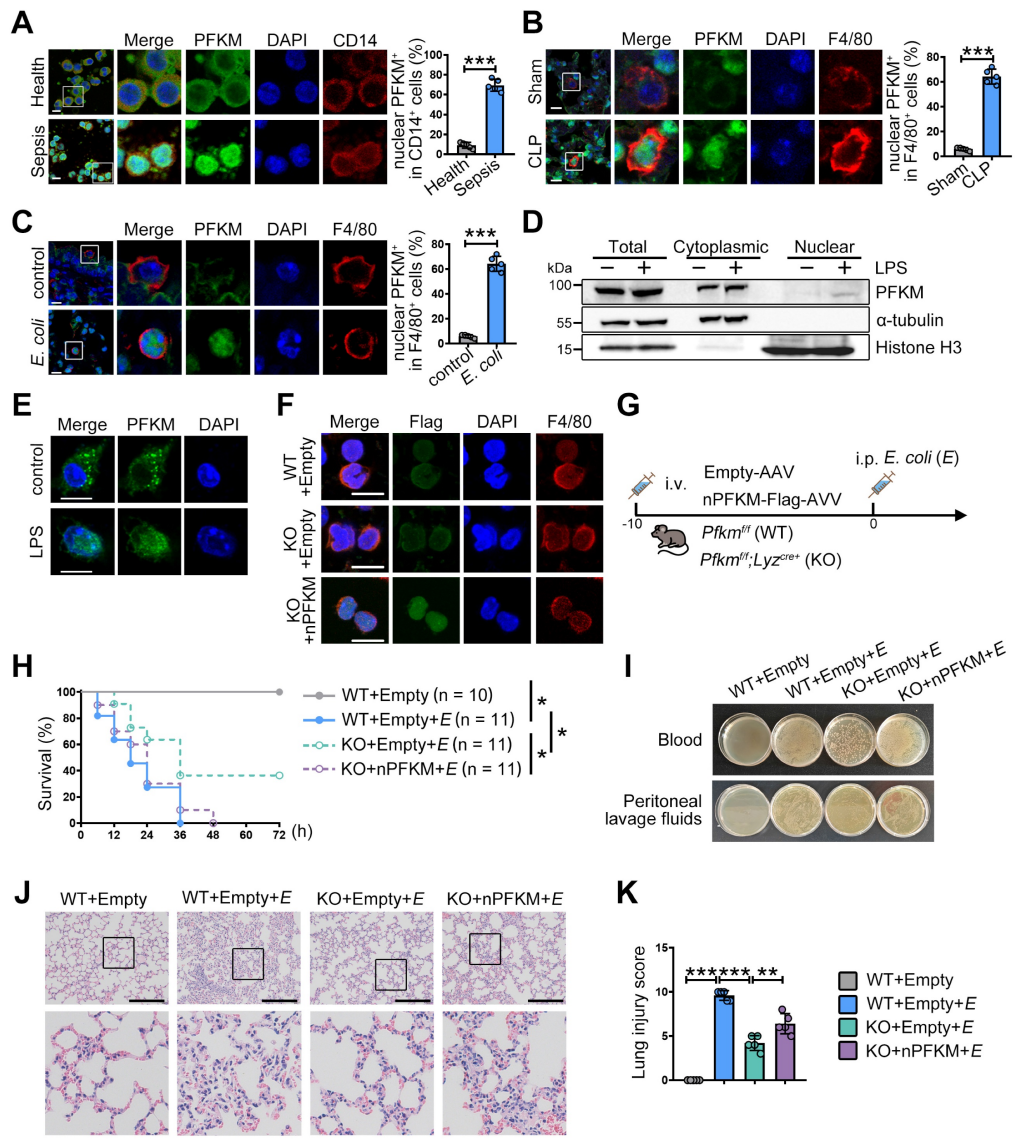
** PFKM accumulating in macrophage nuclei exacerbates sepsis.** (**A-C**) The nuclear translocation ratio of PFKM in CD14^+^ or F4/80^+^ cells was quantified as described in Materials and Methods. (**A**) Representative multiplex immunofluorescent staining of PBMNCs isolated from healthy volunteers and septic patients (n = 5). Scale bars, 10 µm. (**B**) Sepsis was induced by CLP in wild-type (WT) mice. Representative multiplex immunofluorescent staining of lungs was obtained at 24 h post-CLP (n = 5). Scale bars, 10 µm. (**C**) WT mice were injected with *E. coli* (3 × 10⁷ CFU/mouse) intraperitoneally. Representative lung immunofluorescence images at 24 h post-injection (n = 5). Scale bars, 10 µm. (**D-E**) BMDMs from WT mice were stimulated with LPS (100 ng/mL) for 96 h, and the subcellular localization of PFKM was detected by (**D**) Western blotting of cytoplasmic and nuclear fractions and (**E**) immunofluorescence. Scale bars, 10 µm. (**F**) The indicated mouse strains were intravenously injected with adenoviruses (1 × 10^11^ VG/mouse) for 10 days, and representative multiplex immunofluorescent staining of peritoneal macrophages is shown. Scale bars, 10 µm. (**G**) A schematic diagram shows the overexpression of nuclear PFKM (nPFKM) in macrophages, followed by intraperitoneal injection of *E. coli* (E) (5 × 10⁷ CFU/mouse) to induce sepsis in mice. (**H**) Survival curves of mice were recorded (n = 10-11). (**I**) Representative photos of plated blood and peritoneal lavage fluid from mice at 3 h after infection (n = 3). (**J**) Representative H & E staining of lungs at 24 h post-injection (n = 5). Scale bars, 200 µm. (**K**) Histological injury of the lungs was scored (n = 5). Data are expressed as mean ± SD. Statistical significance was determined by unpaired t-test for (A, B, and C), by Mantel-Cox's log-rank test for (H), and by one-way ANOVA for (K). * *P* < 0.05, ** *P* < 0.01, *** *P* < 0.001.

Consistent with our previous study 15, CD14^+^ monocytes from septic patients exhibited elevated PFKM protein levels (**Figure 1A**). Moreover, nuclear translocation of PFKM was detected in approximately 65% of CD14^+^ cells from septic patients (**Figure 1A**). To confirm this, we examined PFKM sub-localization in well-established sepsis mouse models induced by CLP operation or *E. coli* injection. In both models, nuclear accumulation of PFKM occurred in F4/80⁺ macrophages within the lungs (**Figure 1B-C, Movie S1-4**). Furthermore, *in vitro* experiments validated that LPS stimulation triggered nuclear PFKM accumulation in BMDMs (**Figure 1D-E**).

To assess the impact of PFKM nuclear accumulation in macrophages during sepsis, we used myeloid-specific PFKM knockout mice (*Pfkm^f/f^; Lyz^cre+^*, KO). These mice were intravenously injected with adeno-associated virus to overexpress the nuclear PFKM specifically in macrophages (**Figure 1F**), followed by injection with *E. coli* to induce sepsis (**Figure 1G**). PFKM knockout improved survival rates (**Figure 1H**), reduced bacterial loads in blood and peritoneal fluid (**Figure 1I**), and attenuated lung tissue injury (**Figure 1J-K**). Conversely, nuclear PFKM overexpression in macrophages exacerbated sepsis development and reversed these protective effects (**Figure 1H-K**). Collectively, these data indicate that nuclear PFKM enrichment in macrophages promotes sepsis progression, and PFKM nuclear translocation in macrophages could be a risk factor for sepsis.

### Nuclear PFKM accumulation alters macrophage gene profile

**Figure 2:**
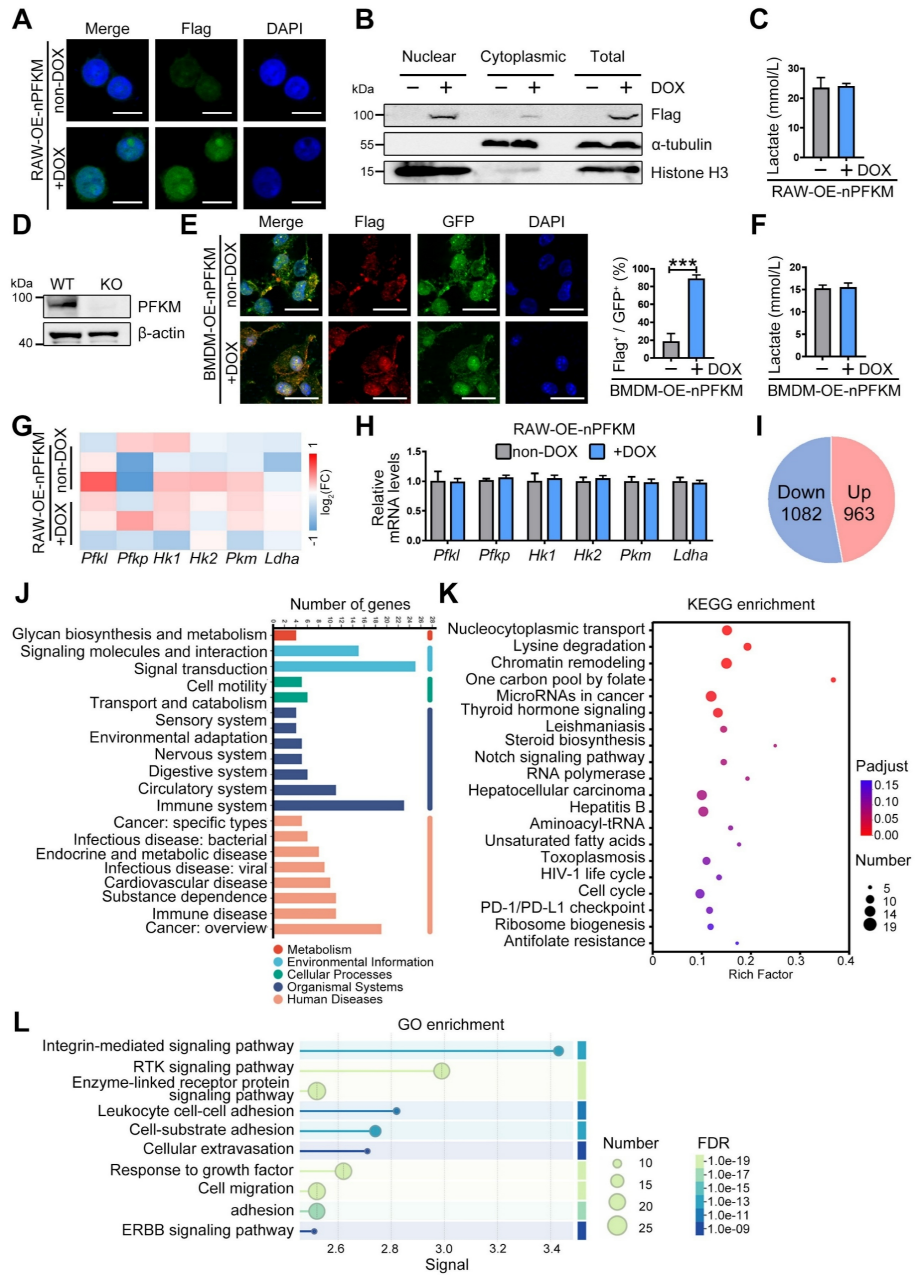
** Overexpressing nuclear PFKM affects the gene profile of macrophages but not glycolysis.** (**A-C**) The RAW-OE-nPFKM cells were established and treated with or without DOX (600 ng/mL) for 48 h. (**A**) Representative multiplex immunofluorescent staining and (**B**) Western blotting analysis was utilized to detect the nuclear PFKM levels. Scale bars, 10 µm. (**C**) Lactate levels in the culture medium of cells were detected by the Lactate Assay Kit. (**D**) Western blotting analysis was utilized to detect the PFKM expression in BMDMs. (**E**) BMDMs from KO mice were infected with both nuclear PFKM and GFP lentivirus, followed by DOX (600 ng/mL) treatment for 48 h. GFP^+^ cells were sorted by flow cytometry. Representative immunofluorescence staining. Statistical data of the proportion of Flag^+^ cells among GFP^+^ cells. Scale bars, 10 µm. (**F**) Lactate levels in the culture medium of cells were measured by the Lactate Assay Kit. (**G**) Heatmap analysis of the key glycolytic kinases and lactate dehydrogenase. (**H**) mRNA expressions of the glycolytic enzymes were detected by real-time RT-PCR. (**I**) The number of up-regulated and down-regulated genes in transcriptomic sequencing. (**J**) Reactome analysis of all differentially expressed genes. (**K**) KEGG enrichment analysis of upregulated genes. (**L**) GO enrichment analysis of upregulated genes. Data are expressed as mean ± SD. Statistical significance was determined by unpaired t-test for (C, E, F, and H). *** *P* < 0.001.

Although PFKM is a rate-limiting enzyme in glycolysis, ectopic expression of nuclear PFKM in the macrophage cell line RAW264.7 cells (RAW-OE-nPFKM) did not alter glycolytic activity as expectedly (**Figure 2A-C**). To validate this, we isolated BMDMs from *Pfkm^f/f^; Lyz^cre+^* mice (**Figure 2D**). Nuclear PFKM overexpression also barely affected the lactate levels of BMDMs (**Figure 2E-F**). Then, we performed transcriptomic sequencing to investigate the gene expression profile in macrophages with ectopic expression of nuclear PFKM. Consistent with the *in vitro* experiment, the mRNA expression of key glycolytic enzymes, including *Pfk1*, *Pfkp*, *Hk1*, *Hk2*, *Pkm,* and *Ldha,* remained stable after ectopically expressed nuclear PFKM (**Figure 2G-H**). Our results demonstrate that nuclear PFKM could mediate macrophage function in a glycolysis-independent manner.

Reactome analysis based on transcriptomic sequencing indicated that the signal transduction and the immune system were enriched (**Figure 2J**). Up-regulated signals in macrophages overexpressing nuclear PFKM (**Figure 2I**) were analyzed using the KEGG pathway database. Data showed that the nucleocytoplasmic transport, chromatin remodeling, and PD-1/PD-L1 checkpoint that influence phagocytosis 24,25 were enriched (**Figure 2K**). In addition, GO enrichment analysis highlighted the signaling pathways associated with macrophage phagocytosis (**Figure 2L**), suggesting that nuclear PFKM could affect phagocytosis of macrophages.

### Nuclear PFKM overexpression impairs macrophage phagocytosis

**Figure 3:**
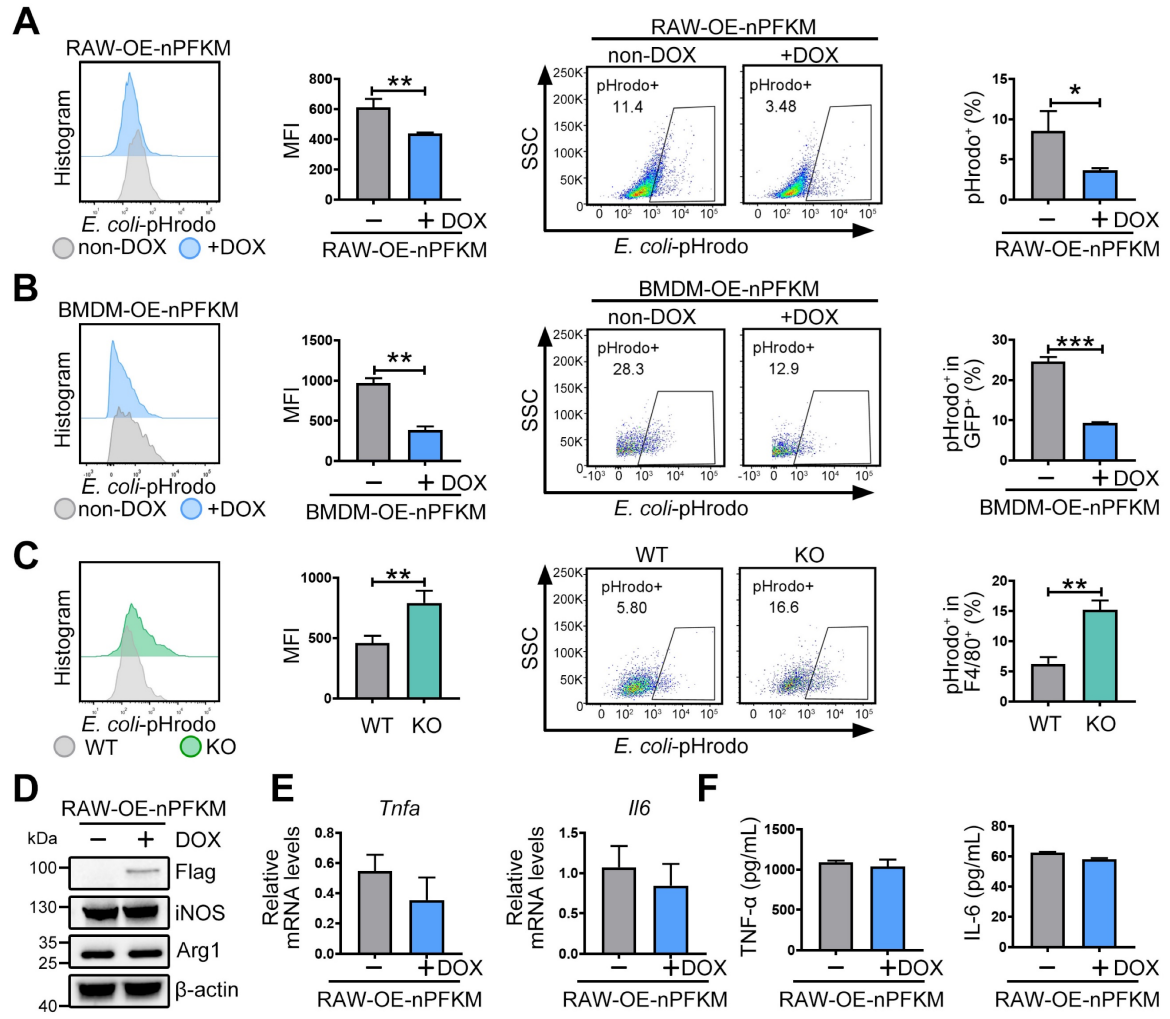
** The effect of nuclear PFKM on macrophage function.** (**A**) RAW-OE-nPFKM cells were treated with or without DOX (600 ng/mL) for 48 h, and then co-cultured with *E. coli*-pHrodo for 1 h. Phagocytosis was assessed by flow cytometry. (**B**) BMDMs from KO mice were infected with nuclear PFKM lentivirus and followed by treatment with or without DOX (600 ng/mL) for 48 h, and then co-cultured with *E. coli*-pHrodo for 1 h. Phagocytosis was assessed by flow cytometry. (**C**) Peritoneal macrophages from WT or KO mice were co-cultured with *E. coli*-pHrodo for 1 h, and phagocytosis was assessed by flow cytometry. (**D-F**) RAW-OE-nPFKM cells were treated with or without DOX (600 ng/mL) for 48 h. (**D**) The protein levels of iNOS and Arg-1 were assessed by Western blotting analysis. (**E**) The mRNA levels of *Tnfa* and *Il6* were measured by real-time RT-PCR. (**F**) The levels of TNF-α and IL-6 in the culture medium were detected using ELISA. Data are expressed as mean ± SD. Statistical significance was determined by unpaired t-test for (A, B, C, E and F). *P < 0.05, **P < 0.01, ***P < 0.001.

To validate whether nuclear PFKM could regulate the macrophage phagocytosis, *E. coli* was labeled with pHrodo and then co-cultured with macrophages. Ectopic expression of nuclear PFKM suppressed bacterial phagocytosis in RAW264.7 cells (**Figure 3A**). Similarly, overexpression of nuclear PFKM impaired BMDM phagocytosis (**Figure 3B**), while *Pfkm* deficiency enhanced the bacterial phagocytosis (**Figure 3C**). Notably, without additional stimulation, nuclear PFKM overexpression did not affect the levels of molecules involved in macrophage polarization (**Figure 3D**) or inflammatory cytokine production (**Figure 3E-F, Figure S1**). Collectively, our results confirm that nuclear PFKM mainly regulates phagocytosis in macrophages.

### Nuclear translocation of PFKM is dependent on p53

**Figure 4:**
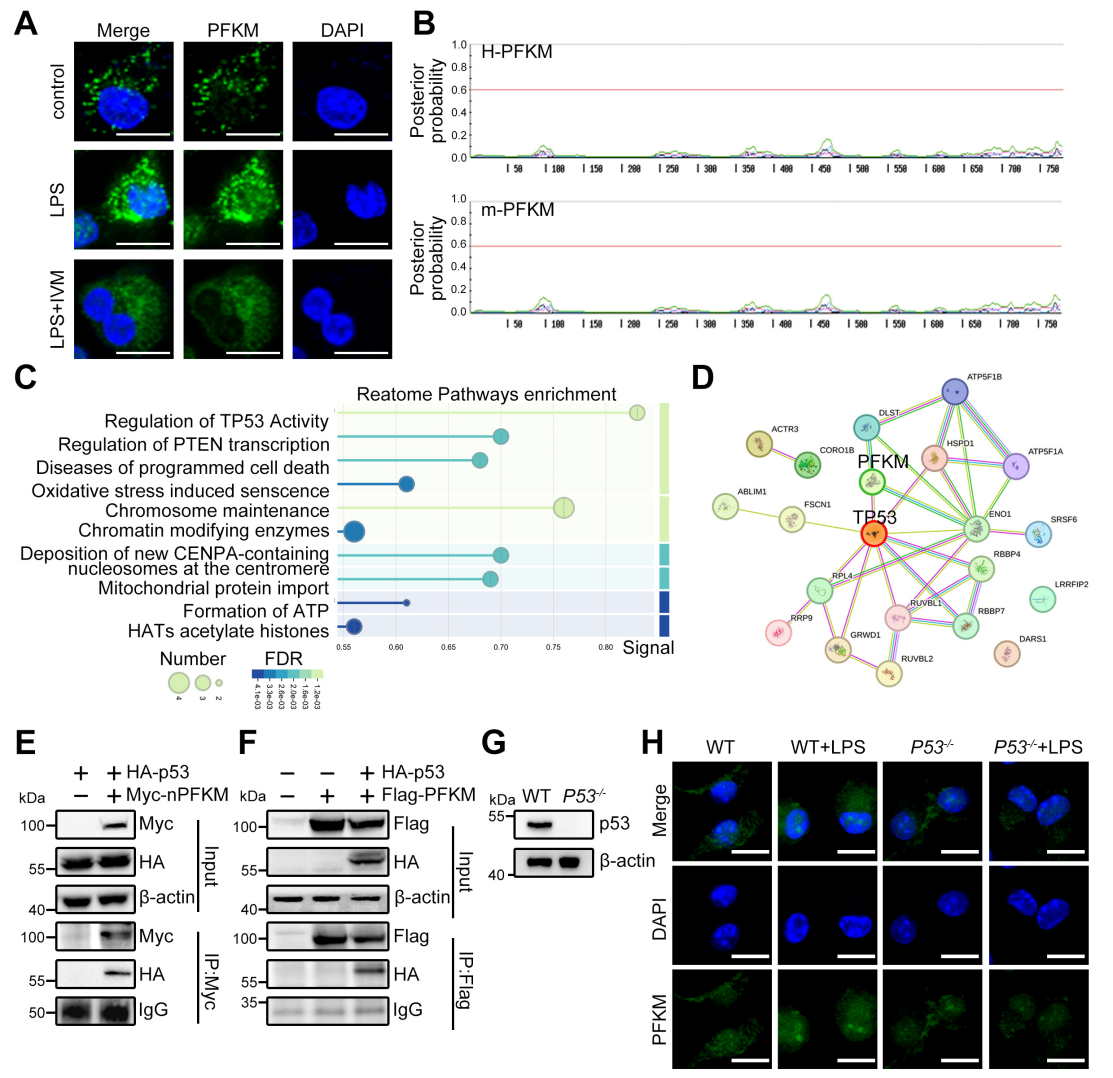
** p53 is crucial for the nuclear translocation of PFKM.** (**A**) Representative immunofluorescent staining of PFKM in WT BMDMs treated with LPS (100 ng/mL) alone or combined with IVM (5 μM) for 96 h. Scale bars, 10 µm. (**B**) The NLStradamus was used to predict the nuclear localization sequence of PFKM (www.moseslab.csb.utoronto.ca/NLStradamus/). (**C**) Proteins interacting with PFKM were analyzed by LC-MS/MS. (**D**) The interaction between PFKM and p53 was predicted using the STRING protein interaction database (string-db.org). (**E-F**) Co-IP combined with Western blotting analysis was used to analyze the interaction of PFKM and p53. (**G**) Western blotting of p53 in BMDMs from WT and p53 knockout mice (*P53^-/-^*). (**H**) Representative immunofluorescent staining of BMDMs stimulated with or without LPS (100 ng/mL) for 96 h. Scale bars, 10 µm.

PFKM is mainly localized to the cytoplasm in cells without stimulation (https://www.proteinatlas.org), yet how PFKM translocates into the nucleus remains unknown. Nuclear import of most proteins necessitates an NLS for specific recognition and transport by importins 26,27. The importin inhibitor IVM reduced LPS-induced nuclear PFKM accumulation (**Figure 4A**). However, no NLS was predicted in PFKM according to its amino acid sequence (**Figure 4B**), demonstrating that PFKM could be indirectly shuttled into the nucleus via other protein(s) containing NLS.

To further validate whether the translocation of PFKM depends on binding to a protein containing NLS, Co-IP/MS was performed and identified p53 as the primary carrier candidate (**Figure 4C**), a famous protein known to harbor a canonical NLS 28. The STRING database predicted the interaction between PFKM and p53 (**Figure 4D**). Furthermore, Co-IP also confirmed that PFKM can bind to p53 (**Figure 4E-F**), and immunofluorescence demonstrated their nuclear co-localization in septic macrophages (**Figure S2, Movie S5-6**). Moreover, we treated BMDM from *P53^-/-^* mice (**Figure 4G**) with LPS, and found that the nuclear accumulation of PFKM was abolished in p53-deficient BMDMs upon LPS stimulation (**Figure 4H**). Collectively, our data reveal that PFKM depends on binding to p53 for its nuclear import.

### Nuclear PFKM promotes PD-1 expression by facilitating p53 acetylation

**Figure 5:**
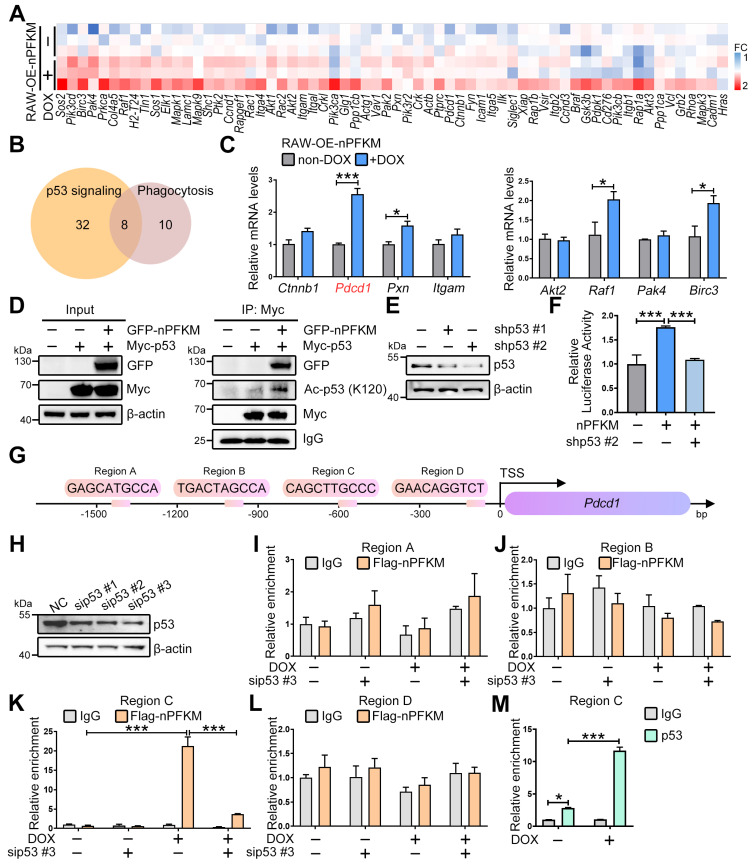
** PFKM-p53 interaction induces PD-1.** (**A**) Heatmap analysis of genes in the Integrin-mediated signaling pathway. (**B**) A Venn diagram shows the intersection of two gene sets: List1 (yellow) represents genes associated with the p53 signaling pathway; List2 (pink) includes genes related to macrophage phagocytosis. (**C**) Real-time RT-PCR shows mRNA expression levels of the 8 indicated genes. (**D**) Co-IP combined with Western Blotting shows the acetylation level of lysine 120 on p53. (**E**) HEK-293T cells were transfected with p53-targeting shRNA (shp53) for 24 h. The p53 level was determined by Western blotting. (**F**) Luciferase assay evaluates the transcriptional activity of *Pdcd1* following nuclear PFKM overexpression and p53 knockdown. (**G**) Schematic diagram of the mouse *Pdcd1* gene locus with four potential p53-binding regions. TSS, transcription start site. (**H**) RAW264.7 cells were transfected with p53-targeting siRNA (sip53) or negative control siRNA (NC) for 24 h. The p53 level was determined by Western blotting. (**I-M**) ChIP analysis of p53 occupancy on *Pdcd1* promoter region in RAW-OE-nPFKM cells treated with or without DOX (600 ng/mL) for 48 h. Enrichment of the *Pdcd1* promoter region was quantified by real-time PCR. (**I-L**) ChIP was performed with an anti-Flag antibody. (**M**) ChIP was performed with an anti-p53 antibody. Data are expressed as mean ± SD. Statistical significance was determined by unpaired t-test for (C), by one-way ANOVA for (F), and by two-way ANOVA for (I, J, K, L, and M). *P < 0.05, ***P < 0.001.

To elucidate the mechanism by which the PFKM-p53 axis suppresses macrophage phagocytosis, we analyzed our transcriptomic data and identified candidate downstream genes from the top Gene Ontology (GO) term (**Figure 5A**). Cross-referencing with the STRING database revealed 8 genes implicated in both p53 signaling and phagocytosis (**Figure 5B**). Among these, *Pdcd1* was the most up-regulated upon nuclear PFKM overexpression (**Figure 5C**), suggesting that PD-1 could function as a key downstream of the PFKM-p53 axis in regulating phagocytosis.

A previous study demonstrated that acetyltransferases p300, CBP, and TIP60 facilitated p53-mediated *Pdcd1* transcription via boosting p53 acetylation at K120 29. To investigate how nuclear PFKM orchestrates p53 to drive *Pdcd1* transcription, we performed Co-IP and ChIP assays. Nuclear PFKM overexpression significantly enhanced p53-K120 acetylation (**Figure 5D**), while this enhancement was abolished upon inhibition of p300/CBP or TIP60 (**Figure S3**). ChIP assays demonstrated specific recruitment of the nuclear PFKM-p53 complex to a region ~0.6 kb upstream (Region C) of the *Pdcd1* promoter (**Figure 5G-M**). The enrichment was strengthened by nuclear PFKM overexpression, while abrogated by p53 knockdown (**Figure 5K**). Consistently, overexpressing nuclear PFKM obviously boosted p53-mediated *Pdcd1* transcription (**Figure 5E-F**). Collectively, our results indicate that nuclear PFKM binding facilitates the recruitment of acetyltransferases to enhance K120 acetylation of p53, thereby promoting p53 transcriptional activity at the *Pdcd1* promoter.

### Nuclear PFKM promotes PD-1 expression to inhibit macrophage phagocytosis

**Figure 6:**
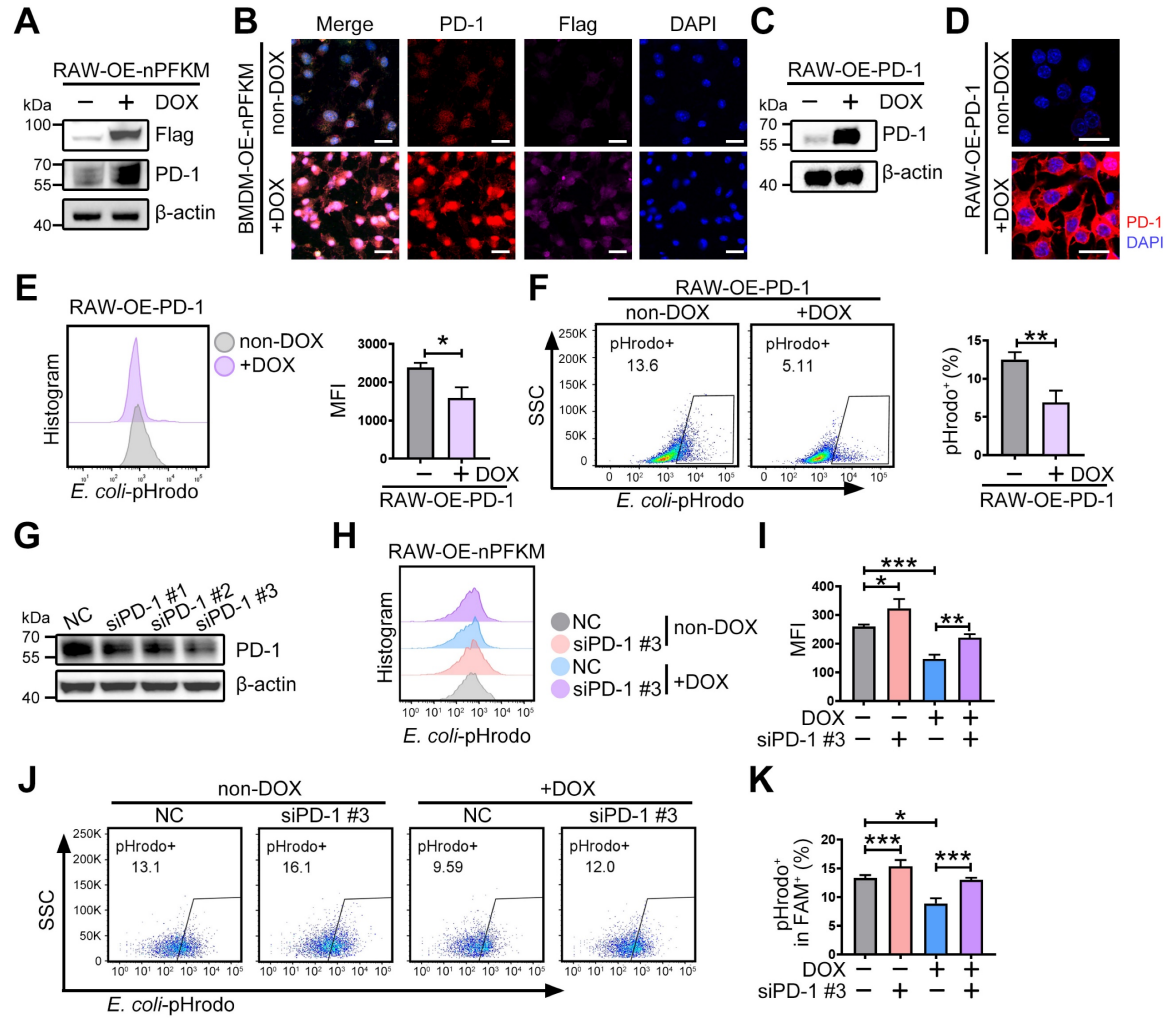
** Nuclear PFKM boosts PD-1 expression and impairs macrophage phagocytosis.** (**A-B**) After treating RAW-OE-nPFKM and BMDM-OE-nPFKM cells with or without DOX (600 ng/mL) for 48 h, the protein expression level of PD-1 was detected by (**A**) Western blotting analysis and (**B**) multiplex immunofluorescence. Scale bars, 20 µm. (**C-D**) RAW264.7-OE-PD-1 cells were treated with or without DOX (300 ng/mL) for 48 h, followed by validation via (**C**) Western blotting analysis and (**D**) immunofluorescence. Scale bars, 20 µm. (**E-F**) RAW264.7-OE-PD-1 cells were treated with or without DOX (300 ng/mL) for 48 h, and then co-cultured with *E. coli*-pHrodo for 1 h. The phagocytosis was assessed by flow cytometry. (**G**) RAW264.7 cells were transfected with PD1-targeting siRNA (siPD-1) or negative control siRNA for 24 h. The protein level of PD-1 was determined by Western blotting analysis. (**H-K**) After treating RAW-OE-nPFKM cells with or without DOX (600 ng/mL) for 48 h, the cells were transfected with PD-1 siRNA or a negative control siRNA for 24 h, followed by co-culture with *E. coli*-pHrodo for 1 h. The phagocytosis was assessed by flow cytometry. Data are expressed as mean ± SD. Statistical significance was determined by unpaired t-test for (E and F) and by one-way ANOVA for (I and K). * *P* < 0.05, ** *P* < 0.01, *** *P* < 0.001.

PD-1 provided negative feedback to macrophages that suppressed phagocytosis 30,31. Therefore, to explore whether nuclear PFKM inhibits macrophage phagocytosis via up-regulating PD-1, we examined the PD-1 protein upon nuclear PFKM overexpression. Overexpression of nuclear PFKM resulted in an elevation of PD-1 protein expression in both RAW264.7 cells and BMDMs (**Figure 6A-B, Figure S4A**). Overexpression of PD-1 (**Figure 6C-D**) suppressed the phagocytosis of macrophages (**Figure 6E-F**), while PD-1 silencing rescued the phagocytosis inhibited by nuclear PFKM in macrophages (**Figure 6G-K**). These results confirm that PD-1 is the downstream effector of the PFKM-p53 axis in regulating macrophage phagocytosis.

### Nanobody Nb07 enhances macrophage phagocytosis by blocking the PFKM-p53 interaction

**Figure 7:**
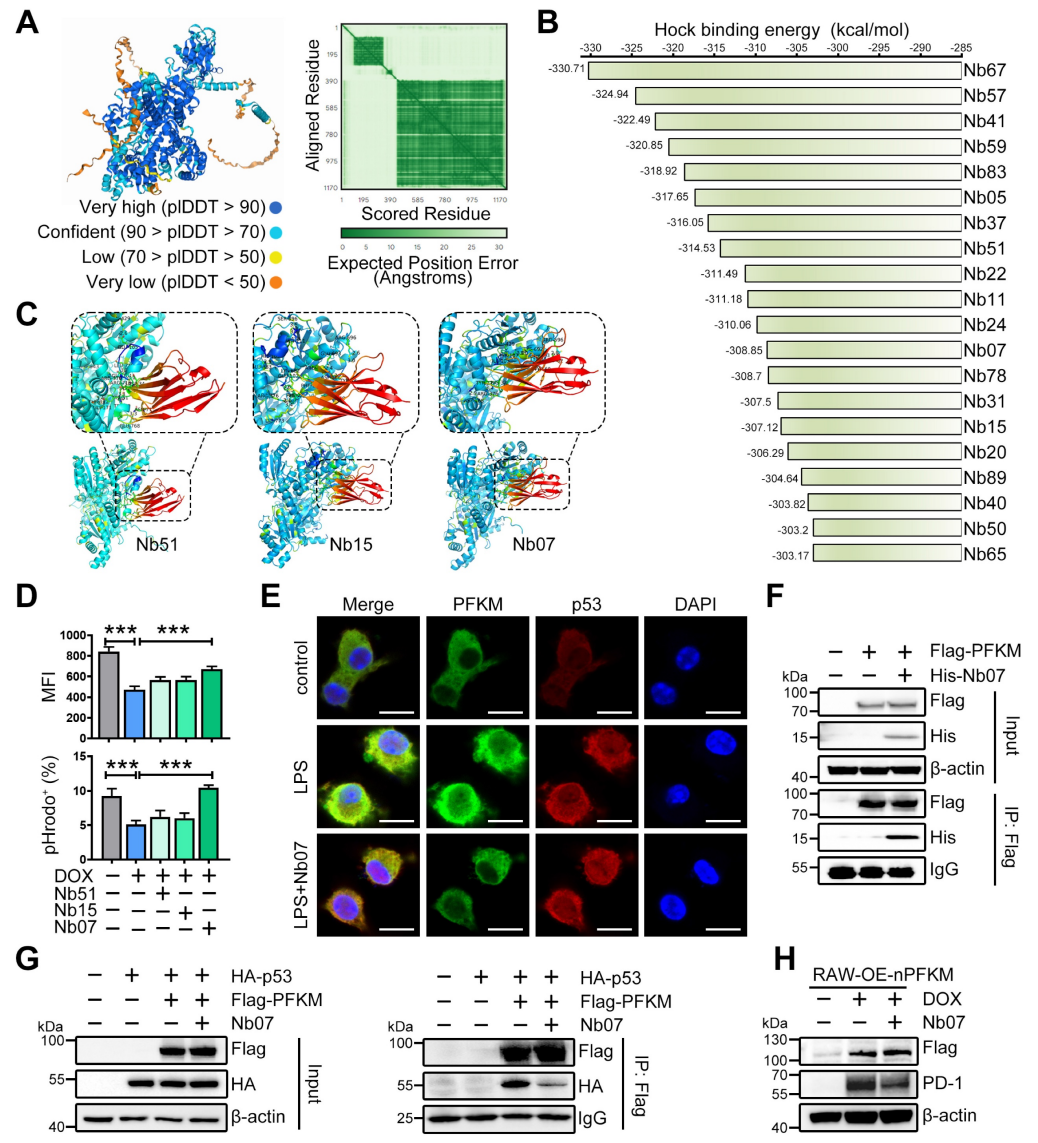
** Nb07 can block the PFKM-p53 interaction.** (**A**) AlphaFold3 predicts the binding sites between PFKM and p53. (**B**) The top 20 affinity of nanobodies for blocking PFKM-p53 interaction. (**C**) Molecular docking of PFKM and separated nanobodies predicts the binding sites of Nb51, Nb15, and Nb07 with PFKM. (**D**) RAW-OE-nPFKM cells were treated with or without DOX (600 ng/mL) for 24 h, then co-treated with Nb51, Nb15, or Nb07 (300 ng/mL) for another 24 h, and then co-cultured with *E. coli*-pHrodo for 1 h. Phagocytosis was assessed by flow cytometry. (**E**) Representative immunofluorescent staining of BMDMs stimulated with or without LPS (100 ng/mL) for 96 h, with treatment with Nb07 (300 ng/mL) for another 24 h. Scale bars, 10 µm. (**F**) Co-IP and Western blotting analyze PFKM-Nb07 binding. (**G**) Co-IP and Western blotting analyze PFKM-p53 binding after Nb07 treatment (300 ng/mL) for 24 h. (**H**) RAW-OE-nPFKM cells were treated with or without DOX (600 ng/mL) for 24 h, then co-treated with Nb07 (300 ng/mL) for another 24 h. Western blotting analysis of PD-1 levels. Data are expressed as mean ± SD. Statistical significance was determined by one-way ANOVA for (D). *** *P* < 0.001.

To develop the strategies for blocking the interaction of PFKM and p53, the PFKM-p53 protein interaction regions were predicted by AlphaFold3. High-confidence regions (pLDDT > 90) were selected (**Figure 7A, Figure S5A**), and virtual screening was then performed to identify single-domain antibodies (or nanobodies) with optimal structural compatibility. From the top 20 sequences with the highest predicted binding affinity to PFKM (**Figure 7B**), we selected Nb51, Nb15, and Nb07 for synthesis based on their capacity to occupy most of the PFKM-p53 binding surface (**Figure 7C**). Functional testing demonstrated that Nb51 or Nb15 only slightly improved macrophage phagocytosis, while Nb07 significantly restored the macrophage phagocytic capacity upon ectopic expression of nuclear PFKM (**Figure 7D**).

Nb07 only comprises a single heavy-chain variable domain with a molecular weight of approximately 14 kDa (**Figure S5C**), and a hydration particle size of 260.4 nm (**Figure S5D**). Nb07 presented low cytotoxicity (**Figure S5E-F**), and both *in vivo* and *in vitro* experiments validated that Nb07 was efficiently internalized by macrophages (**Figure S5G-H**).

To investigate whether Nb07 could impair the nuclear translocation of PFKM by disrupting PFKM-p53 binding, we treated BMDMs with LPS in the presence or absence of Nb07. Nb07 treatment decreased the LPS-induced nuclear accumulation of PFKM without affecting p53 nuclear localization (**Figure 7E**). Co-IP assay and SPR experiments verified that Nb07 can directly bind to PFKM (**Figure 7F, Figure S5B, Table S5**), thereby reducing PFKM interaction with p53 (**Figure 7G**), which subsequently abrogated nuclear PFKM-induced PD-1 up-regulation (**Figure 7H**). In addition, Nb07 treatment decreased LPS-induced PD-1 expression in BMDMs (**Figure S6A**). These data indicate that Nb07 could enhance macrophage phagocytosis by inhibiting the PFKM-p53-PD-1 axis, highlighting its potential as a therapeutic strategy against sepsis.

### Nanobody Nb07 alleviates sepsis

**Figure 8:**
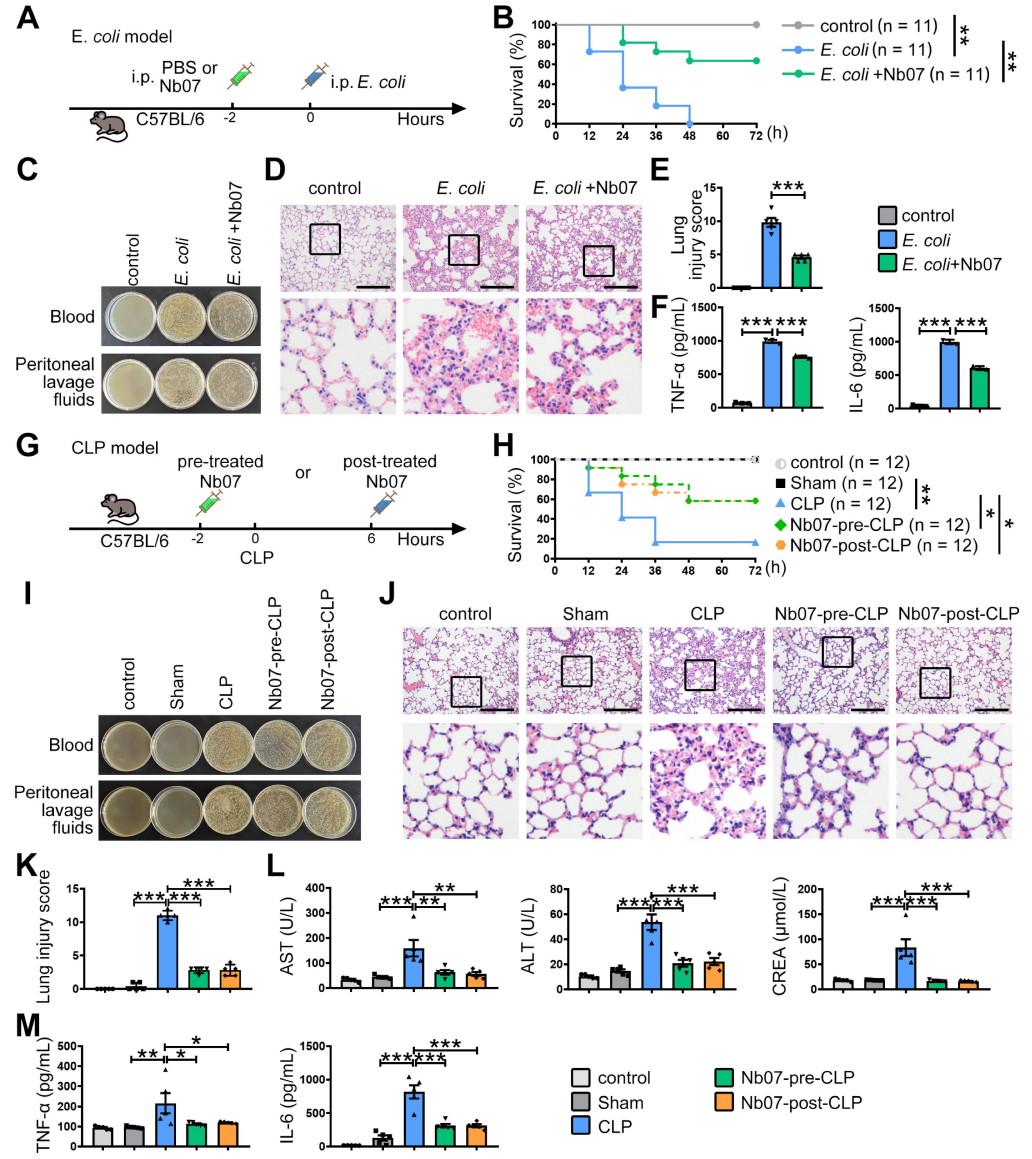
** The nanobody Nb07 has the potential to mitigate sepsis.** (**A**) Schematic diagram of the experiment: Mice were injected with Nb07 (1 mg/kg) or PBS 2 h before intraperitoneal injection of *E. coli* (3 × 10⁷ CFU/mouse). (**B**) Survival curves of mice were recorded (n = 11). (**C**) Representative photos of plated blood and peritoneal lavage fluid from mice at 3 h after infection (n = 3). (**D-E**) Representative H & E staining of lungs at 24 h post-injection, and histological injury of the lungs was scored (n = 5). Scale bars, 200 µm. (**F**) Peripheral blood was obtained at 3 h, and the levels of TNF-α and IL-6 were measured by ELISA. (**G**) Schematic diagram of the experiment: Mice were intraperitoneally injected with Nb07 2 h before CLP operation or 6 h post-CLP operation. (**H**) Survival curves of mice were recorded every 12 h (n = 12). (**I-M**) Mice were sacrificed at 24 h (n = 5). (**I**) Representative photos of plated blood and peritoneal lavage fluid. (**J**) Representative H & E staining of lungs and (**K**) histological injury was scored. Scale bars, 200 µm. (**L**) The levels of AST, ALT, and CREA in serum were detected by an automatic biochemical analyzer. (**M**) The levels of TNF-α and IL-6 in serum were measured by ELISA. Data are expressed as mean ± SD. Statistical significance was determined by one-way ANOVA for (E-F) and (K-M) and by Mantel-Cox's log-rank test for (B) and (H). * *P* < 0.05, ** *P* < 0.01, *** *P* < 0.001.

Finally, to evaluate the preventive effect of Nb07, mice were treated with Nb07 or PBS before intraperitoneal injection of *E. coli* to induce sepsis (**Figure 8A**). Nb07 treatment improved the survival rate of septic mice (**Figure 8B**), reduced bacterial loads in blood and peritoneal fluid (**Figure 8C**), and attenuated pathological features in lung tissues, including alveolar wall thickening, hyperemia, and inflammatory exudation (**Figure 8D-E**). Also, Nb07 treatment reduced the serum levels of TNF-α and IL-6 (**Figure 8F**). These data reveal that Nb07 can confer protection against *E. coli*-induced sepsis.

To further verify the therapeutic effect of Nb07, we used the well-established CLP-induced sepsis model. Mice received Nb07 either 2 h before (pre-treatment) or 6 h after (post-treatment) the CLP procedure (**Figure 8G**). Both pre-treatment and post-treatment improved the survival rate to 60% (**Figure 8H**). Compared with the CLP group, mice in both pre-treatment and post-treatment groups had less bacterial burden in the blood and peritoneum (**Figure 8I**). Nb07 treatment also alleviated the lung injury (**Figure 8J-K**) and reduced serum levels of the liver damage markers, aspartate aminotransferase (AST) and alanine aminotransferase (ALT), and kidney damage marker creatinine (CREA) (**Figure 8L**). Also, Nb07 treatment reduced the serum levels of TNF-α and IL-6 (**Figure 8M**). Additionally, Nb07 treatment also reduced the PD-1 expression in F4/80^+^ lung macrophages (**Figure S6B**). Taken together, our data indicate that Nb07 enhances bacterial clearance and mitigates sepsis in mice.

## Discussion

Here, we demonstrate that infection-induced nuclear translocation of PFKM in macrophages impaired phagocytosis by enhancing p53-mediated PD-1 expression, thereby promoting sepsis progression. This impairment was reversed by Nb07, which blocked PFKM nuclear localization (**Figure 7E**) and mitigated sepsis development (**Figure 8**). Our findings highlight the therapeutic potential of targeting macrophage PFKM in sepsis.

PFKM deletion in myeloid cells mitigates sepsis development 15. Although neutrophils and macrophages are the most abundant myeloid cell populations in early sepsis, RNA and protein analyses demonstrated that PFKM expression was higher in macrophages than in neutrophils (www.proteinatlas.org, **Figure S7**), suggesting that nuclear PFKM mainly affects macrophages during sepsis.

We confirmed that PFKM underwent nuclear translocation by binding to p53 (**Figure 4**). During sepsis, p53 indeed localized to the nucleus in F4/80^+^ macrophages (**Figure S2, Movie S5-6**). As a transcription factor, p53 is known to enter the nucleus under various stress conditions 32-34. Notably, while stressors such as LPS, 5-FU, and ultraviolet irradiation all induced p53 nuclear accumulation and elevated PFKM protein levels, only LPS stimulation triggered the concurrent nuclear translocation of PFKM (**Figure 7E, Figure S8**). This indicates that PFKM-p53 nuclear translocation may be a sepsis-specific response.

The nuclear PFKM-p53 interaction promoted K120 acetylation of p53 (**Figure 5D**), a modification known to enhance its transcriptional activity and mediated by acetyltransferases CBP, p300, and Tip60 29,35-37. Nuclear PFKM overexpression slightly induced the mRNA levels of these acetyltransferases (**Figure S3A**). Moreover, inhibitors of CBP/p300 (C646) or Tip60 (MG149) suppressed the nuclear PFKM-induced p53 acetylation (**Figure S3B**), yet the precise mechanism by which PFKM promotes p53 acetylation requires further investigation.

We identified PD-1 as a key downstream target of the PFKM-p53 pathway. PD-1 expression is up-regulated in monocytes from septic patients 38 and macrophages from septic mice (**Figure S4B, S6B, S9A**), while PD-1 deletion protected mice from sepsis 39. Mechanistically, PD-1 recruits and activates SHP-2 via its intracellular Y248-ITSM motif 40, leading to the dephosphorylation of key phagocytosis-initiating proteins, including Syk (Y352), PI3K-p85α (Y688/Y607), and PLCγ2 (Y753/Y759) 35,41. Furthermore, SHP-2-mediated dephosphorylation of Vav impairs Rac activation and F-actin reorganization, which are essential for phagosome formation 42,43. Nevertheless, these mechanisms are largely derived from studies on tumor cell clearance. How PD-1 regulates macrophage phagocytosis of bacteria during infection remains unclear.

Based on the PFKM-p53-PD-1 axis, we developed the nanobody Nb07 to target the PFKM-p53 interaction. We noted that while Nb07 significantly reduced PFKM-p53 binding, it only partially suppressed PD-1 up-regulation after overexpressing nuclear PFKM (**Figure 7H**), suggesting that additional pathways contribute to PD-1 expression. Researches show that the SMAD3-STAT3 complex and NF-κB response elements can bind to the *Pdcd1* promoter directly and promote its transcription 44,45. Furthermore, inflammatory and metabolic signals also upregulated PD-1 via the mTORC1 pathway 31. These findings imply that PFKM-p53 represents a key, but not exclusive, driver of PD-1 in sepsis. Additionally, p53 can regulate diverse cellular processes, including cell cycle and apoptosis, via regulating a broad spectrum of genes beyond *Pdcd1*. It will be important to investigate whether Nb07 affects the expression of other classical p53 targets, such as CDKN1A (cell cycle), BAX, and PUMA (apoptosis), in future studies.

Unlike the monoclonal antibodies that target PD-1 directly and block its interactions with ligands such as PD-L1 or CD47 46,47, Nb07 acts upstream to restore macrophage phagocytosis (**Figure 7D**). Although PD-1 knockdown enhanced the phagocytosis (**Figure 6G-K**), a PD-1 monoclonal antibody failed to rescue the impaired phagocytosis caused by nuclear PFKM overexpression (**Figure S10**), likely because bacteria lack PD-1 ligands, thereby explaining the limited efficacy of anti-PD-1 antibodies in sepsis clinical trials 48,49. Notably, PFKM deletion reduced PD-1 but not PD-L1 expression in lung F4/80^+^ macrophages from septic mice (**Figure S9**), suggesting that nuclear PFKM regulates macrophage phagocytosis independently of PD-1-PD-L1 interaction. Additionally, compared with the monoclonal antibodies of PD-1 (molecular weight ~ 150 kDa), nanobodies have a smaller molecular weight and size (molecular weight ~ 14 kDa) (**Figure S5C-D, Table S4**), which may confer superior tissue penetration and lower cytotoxicity (**Figure S5E-F**). These properties could highlight the potential advantages of Nb07 in sepsis treatment.

Although Nb07 treatment affected the serum IL-6 and TNF-α levels *in vivo* unexpectedly (**Figure 8**), no such effect was observed in macrophages *in vitro* (**Figure S11A-B**). We speculated that Nb07 enhanced pathogen clearance through improved phagocytosis, thereby reducing the systemic inflammatory response triggered by persistent infection 50. This is consistent with the observation 51 that impaired macrophage phagocytosis tended to be accompanied by elevated serum inflammatory cytokines in bacterial-infected mice (**Figure 3E-F, Figure S12**). It remains possible that the serum inflammatory cytokine levels could be produced by other immune cells* in vivo*.

During our present study, we used the Tet-On system to establish the stable cell lines, which are activated upon DOX treatment. To exclude the possibility that DOX might influence macrophage phagocytosis, we treated RAW264.7 cells with DOX for 48 h. Results showed that DOX treatment did not affect the phagocytosis of macrophages (**Figure S13**).

Our study has several limitations. First, while we identified the PFKM-p53 interaction, the post-translational modifications regulating PFKM nuclear translocation, such as phosphorylation and acetylation, remain unknown. Second, the therapeutic potential of Nb07 in humans is uncertain due to the relatively low homology between murine and human p53 (77.78%), despite high PFKM homology (97.82%). Third, the specific stage of sepsis during which PFKM undergoes nuclear translocation remains undefined, limiting insights into the optimal therapeutic window. Finally, although Nb07 may specifically inhibit genes co-regulated by the PFKM-p53 complex, the full repertoire of targets regulated by this complex remains to be elucidated.

## Conclusion

In summary, we revealed that PFKM bound to and promoted p53 acetylation, enhancing p53-mediated transcriptional activation of *Pdcd1*, which subsequently suppressed macrophage phagocytosis and exacerbated sepsis. The nanobody Nb07 can restore macrophage phagocytosis by blocking PFKM-p53 interaction and ultimately alleviate sepsis, highlighting a novel therapeutic strategy for this condition.

## Supplementary Material

Supplementary movie legends, figures and tables.

Supplementary movie 1.

Supplementary movie 2.

Supplementary movie 3.

Supplementary movie 4.

Supplementary movie 5.

Supplementary movie 6.

## Abbreviations

AAV: Adeno-Associated Virus
Ac: Acetylation
ALT: alanine aminotransferase
Arg1: Arginase 1
AST: aspartate aminotransferase
BMDMs: Bone marrow-derived macrophages
CDR3: Complementarity-Determining Region 3
CFU: Colony-Forming Unit
CLP: Cecal ligation and puncture
Co-IP/MS: Co-immunoprecipitation coupled with mass spectrometry
CREA: Creatinine
DMEM: Dulbecco's Modified Eagle Medium
DOX: Doxycycline
*E*: *E. coli*
ELISA: Enzyme-Linked Immunosorbent Assay
FBS: Fetal Bovine Serum
FITC: fluorescein isothiocyanate
H & E: Hematoxylin and Eosin staining
i.p.: Intra peritoneale
i.v.: Intra venosum
IVM: Ivermectin
KO: *Pfkm^f/f^; Lyz^cre+^*
LPS: Lipopolysaccharides
M-CSF: macrophage colony-stimulating factor
Nb: Nanobody
NLS: Nuclear Localization Signal
nPFKM: Nuclear PFKM
OE: overexpression
PAMPs: The pathogen-associated molecular patterns
PBMNCs: Peripheral blood mononuclear cells
PBS: Phosphate buffer solution
PCR: Polymerase chain reaction
PD-1: Programmed cell death protein 1
PD-L1: Programmed Death-Ligand 1
PFKL: Phosphofructokinase, Liver Type
PFKM: Muscle isoform of phosphofructokinase-1
PFKP: Phosphofructokinase, Platelet type
PFK1: Phosphofructokinase 1
PKM2: Pyruvate Kinase M2
p53: Tumor protein 53
*P53^-/-^*: p53 knockout mice
shp53: p53-targeting shRNA plasmid
siPD-1: PD1-targeting siRNA
sip53: p53-targeting siRNA
SPF: Specific pathogen-free
TAMs: Tumor-associated macrophages
TSS: transcription start site
UV: Ultraviolet radiatio
WT: Wild-type
5-FU: 5-fluorouracil
