# Supplementary material for: Nanobody Nb07 mitigates sepsis by blocking the PFKM-p53-PD-1 axis to enhance macrophage phagocytosis
Source: Theranostics. 2026 Jan 1;16(7):3408–25. doi: 10.7150/thno.124303 (PMC12846728; doi:10.7150/thno.124303)
Supplement: Supplementary file 1 — Supplementary movie legends, figures and tables. [file thnov16p3408s1.pdf]

Supplementary Movies

Movie S1-2, 5-6: Sepsis was induced by CLP in wild-type (WT) mice. Representative multiplex immunofluorescent staining of the lungs was performed at 24 h post-CLP. Confocal microscopy was used to capture Z-stack images, which were then exported as a 3D reconstruction video using ImageJ. S1: sham; S2: CLP; S5: sham; S6: CLP.

Movie S3-4: WT mice were injected intraperitoneally with *E. coli* (3×10^7 CFU/mouse). 24 h later, mice were euthanized, and lung tissues were removed and fixed. Representative images of the lungs were obtained by multiplex immunofluorescent staining. Z-stack images were acquired using confocal microscopy and subsequently processed with ImageJ to generate a 3D reconstruction. S3: control; S4: *E. coli*.

Supplementary Figures

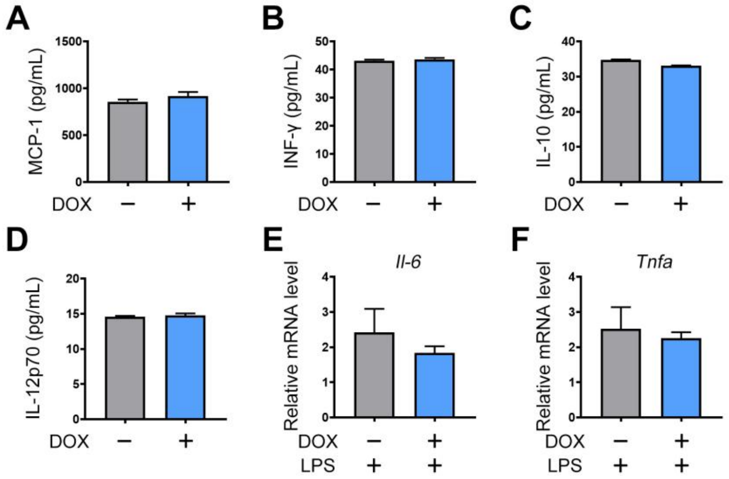

Figure S1: Overexpression of nuclear PFKM in macrophages barely affects the production of inflammatory cytokines.

(**A-D**) RAW-OE-nPFKM cells were treated with or without DOX (600 ng/mL) for 48 h. The CBA kit was used to measure the levels of inflammatory cytokines in the culture medium. (**E-F**) RAW-OE-nPFKM cells were treated with or without DOX (600 ng/mL) for 48 h. Subsequently, the cells were stimulated with LPS (100 ng/mL) for an additional 12 h. The mRNA levels of *Il6* and *Tnfa* were measured by real-time RT-PCR. Data are presented as mean ± SD. Statistical significance was determined by unpaired t-test.

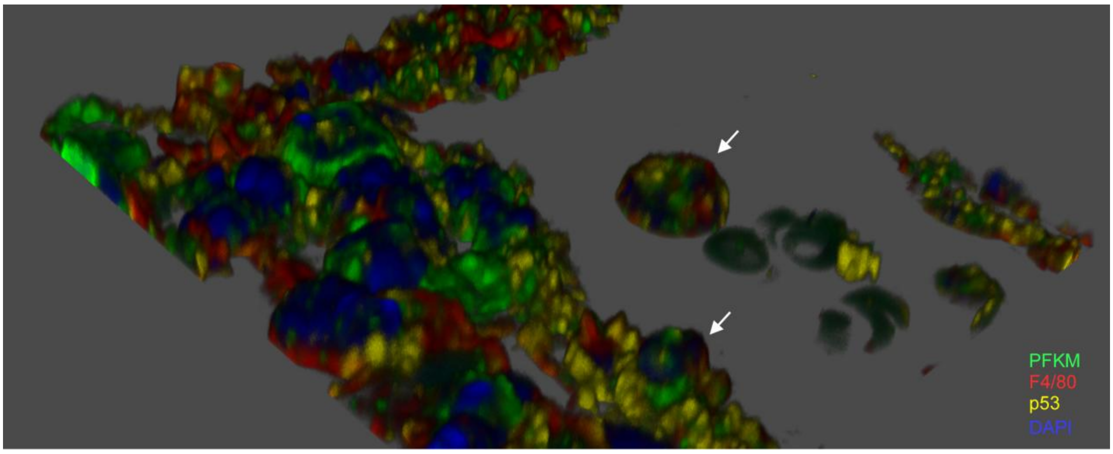

Figure S2: In sepsis, PFKM and p53 co-localize in the nuclei of macrophages.

Sepsis was induced by CLP in WT mice. Representative immunofluorescence images were obtained as z-stacks using a 63 × objective on a confocal microscope and are presented as 3D projections.

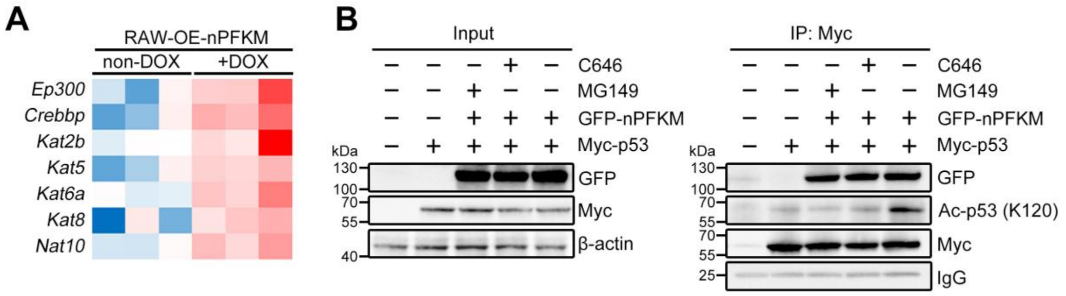

Figure S3: Nuclear PFKM recruits multiple acetyltransferases to promote p53 acetylation modification.

(**A**) Heatmap analysis of acetyltransferases known to modulate p53 acetylation based on transcriptomic sequencing. (**B**) Transfected HEK-293T cells were treated with or without C646 (10 μM) or MG149 (200 μM) for 24 h. Co-IP combined with Western blotting was used to determine the acetylation level of lysine 120 on p53.

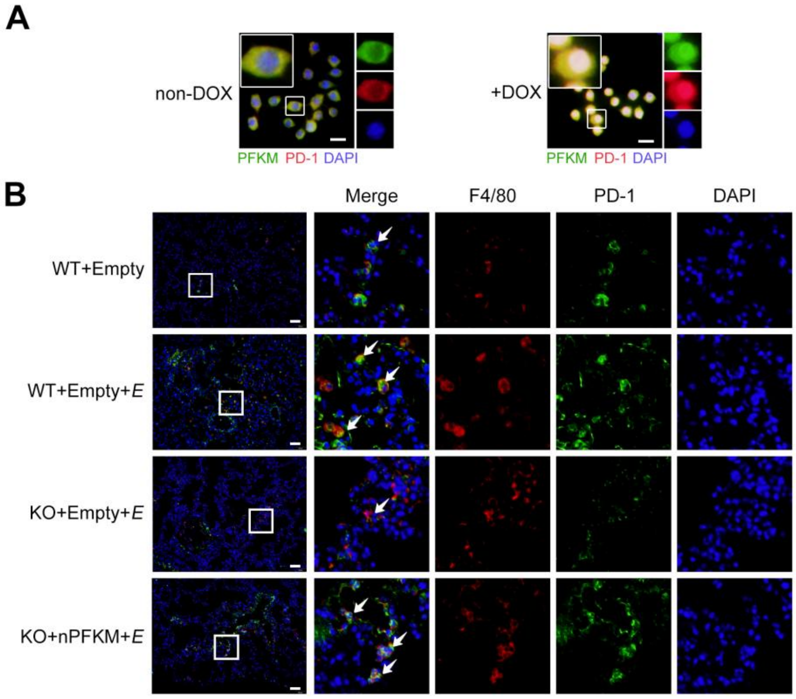

Figure S4: Nuclear PFKM promotes PD-1 expression.

(**A**) RAW-OE-nPFKM cells were treated with or without DOX (600 ng/mL) for 48 h, and the level of PD-1 was detected by multiplex immunofluorescence. Scale bars, 20 μm. (**B**) Mice were injected with adeno-associated virus to overexpress the nuclear PFKM in macrophages, followed by injection with *E. coli* to induce sepsis. Mice were sacrificed at 24 h, and lungs were obtained for multiplex immunofluorescence staining. Scale bars, 50 μm.

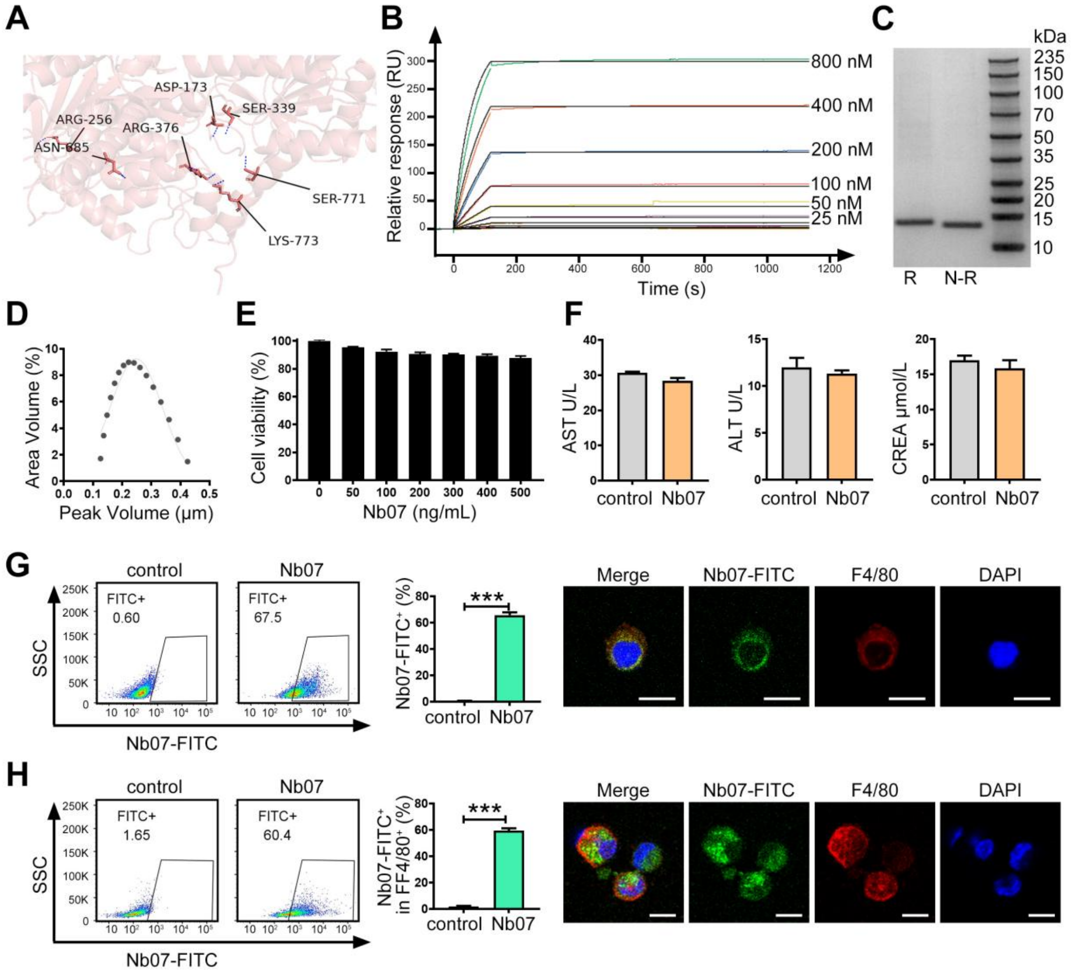

Figure S5: Characteristics of Nb07.

(**A**) Molecular docking predicts the binding sites of PFKM with p53. (**B**) SPR experiments detect the affinity between Nb07 and PFKM (1:1). (**C**) Nb07 was stained with Coomassie Brilliant Blue after SDS-PAGE. (**D**) Particle Size Distribution of Nb07 analyzed using Anton Paar Litesizer 500. (**E**) RAW264.7 cells were treated with Nb07 at different concentrations for 24 h, and the cell viability was determined by the CCK-8 assay. (**F**) Mice were injected with Nb07 (1 mg/kg) or PBS for 24h. The levels of AST, ALT, and CREA in serum were detected by an automatic biochemical analyzer. (**G**) RAW264.7 cells were treated with Nb07-FITC (300 ng/mL) for 24 h, and the uptake was analyzed via flow cytometry and immunofluorescence

staining. Scale bars, 10 μm. (**H**) WT mice were intraperitoneally injected with Nb07-FITC (1 mg/kg) for 24 h, and then the uptake of Nb07 by peritoneal macrophages was analyzed via flow cytometry and immunofluorescence staining. Scale bar, 10 μm. Data are presented as mean ± SD. Statistical significance was determined by unpaired t-test. ***$P < 0.001$.

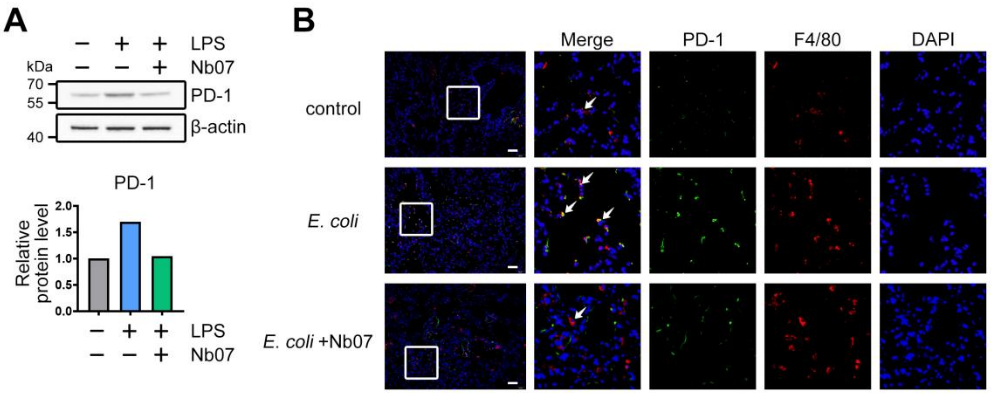

Figure S6: Nb07 reduces PD-1 levels of macrophages.

(**A**) BMDM cells were treated with or without LPS (100 ng/mL) for 96 h, then co-treated with Nb07 (300 ng/mL) for another 24 h. Western blotting analysis of PD-1 levels. (**B**) Mice were intraperitoneally injected with Nb07 (1 mg/kg) or PBS 2 h before intraperitoneal injection of *E. coli* (3×10^7 CFU/mouse). Mice were sacrificed at 24 h to obtain the lungs for multiplex immunofluorescence staining. Scale bars, 10 μm.

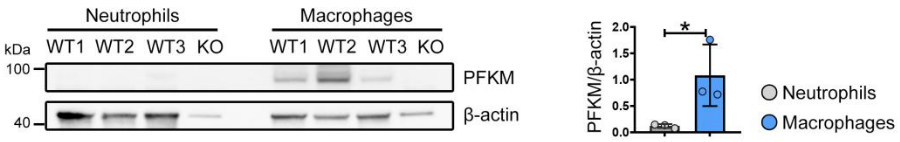

Figure S7: The expression of PFKM in macrophages is significantly higher than that in neutrophils.

Neutrophils and macrophages were sorted from mouse bone marrow using flow cytometry, followed by Western blotting analysis to examine PFKM levels. The density was quantified by the ImageJ software. Data are presented as mean ± SD. Statistical significance was determined by unpaired t-test. *$P < 0.05$.

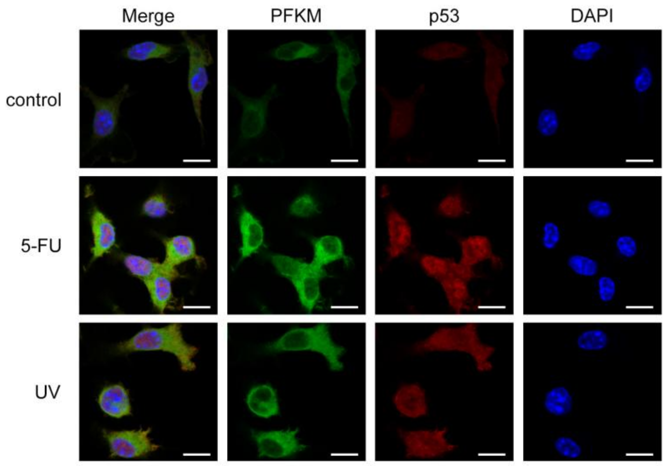

Figure S8: Multiple stressors induce p53 nuclear translocation in macrophages.

BMDMs were treated with 5-fluorouracil (5-FU, 10 μM, 24 h) or UV (50 J/m^2 , 12 h). Representative immunofluorescent staining of PFKM and p53 in WT BMDMs. Scale bars, 10 μm.

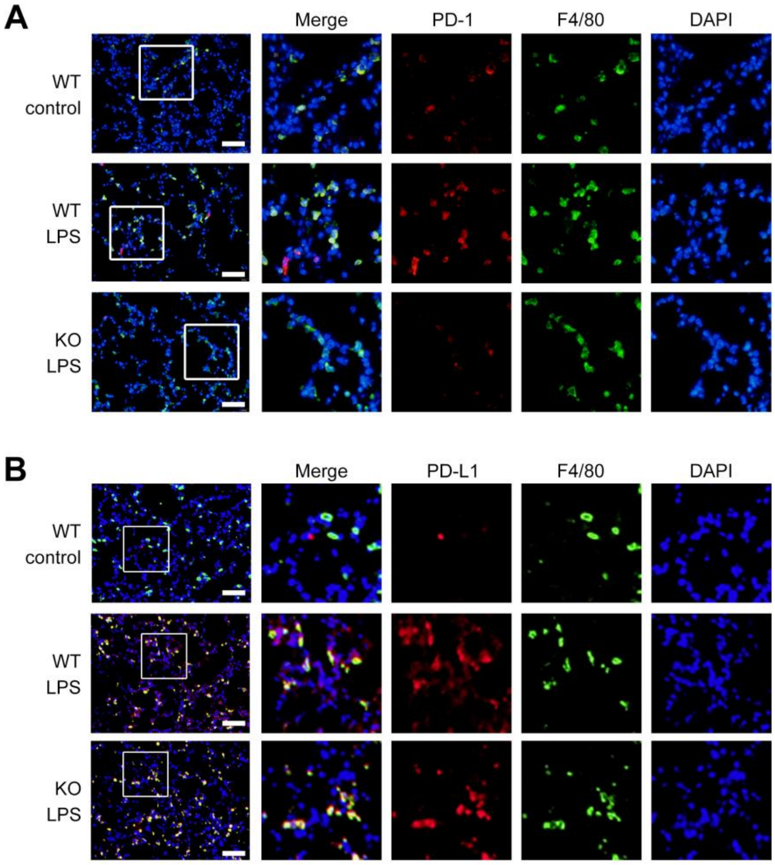

Figure S9: PFKM knockout reduces the protein expression of PD-1 but not PD-L1 in macrophages.

(**A-B**) Mice were intraperitoneally injected with LPS (20 mg/kg) for 24 h, and then the lungs were obtained for multiplex immunofluorescence staining. Scale bars, 50 μm.

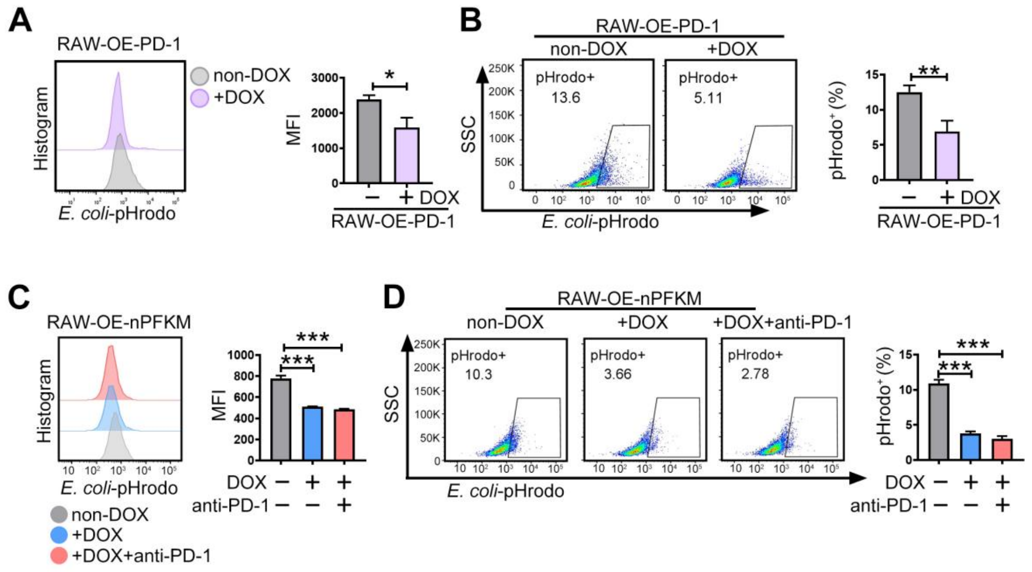

Figure S10: Anti-PD-1 monoclonal antibody is unable to restore the phagocytosis of macrophages.

(**A-D**) RAW-OE-nPFKM cells were treated with or without DOX (600 ng/mL) for 48 h. Subsequently, the cells were cultured in the absence or presence of an anti-PD-1 monoclonal antibody (300 ng/mL) for an additional 24 h. Phagocytosis was assessed using flow cytometry. Data are presented as mean ± SD. Statistical significance was determined by one-way ANOVA. ***P < 0.001.

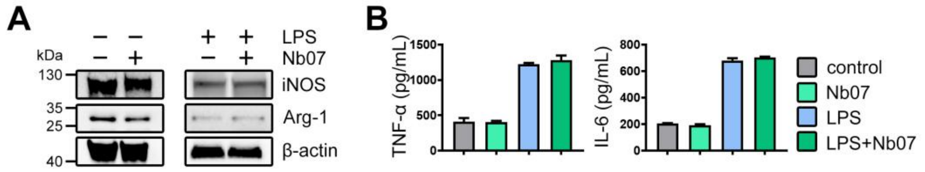

Figure S11: The impact of Nb07 on the levels of inflammatory cytokines and damage markers in macrophages.

(**A-B**) BMDMs were pre-treated with Nb07 (300 ng/mL) for 24 h, then stimulated with or without LPS for 12 h (100 ng/mL). (**A**) The indicated protein was determined using Western blotting analysis, and (**B**) TNF-α and IL-6 levels were measured by ELISA. Data are presented as mean ± SD. Statistical significance was determined by one-way ANOVA.

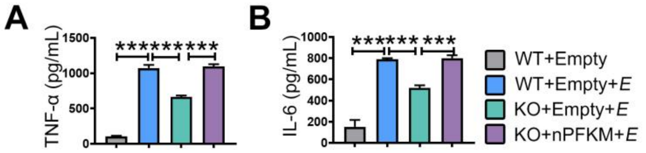

Figure S12: The impact of nuclear PFKM overexpression on the levels of inflammatory cytokines in vivo.

(**A-B**) Mice were injected with adeno-associated virus to over-express the nuclear PFKM in macrophages, followed by injection with a lethal dose of *E. coli* to induce sepsis (5×10^7 CFU/mouse). Peripheral blood was obtained at 3 h, and the levels of TNF-α and IL-6 were measured by ELISA. Data are presented as mean ± SD. Statistical significance was determined by one-way ANOVA. ***$P < 0.001$.

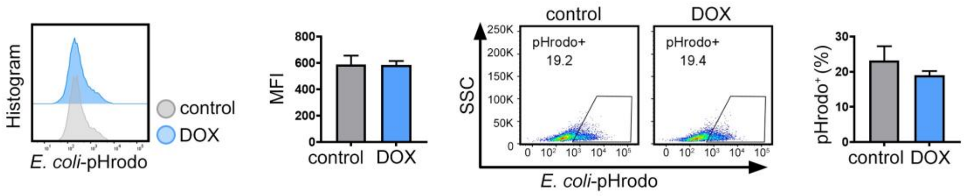

Figure S13: DOX treatment does not affect macrophage phagocytosis.

RAW264.7 cells were treated with DOX (600 ng/mL) for 48 h, and then co-cultured with pHrodo-*E. coli* for 1 h. Phagocytosis was assessed using flow cytometry. Data are presented as mean ± SD. Statistical significance was determined by unpaired t-test.

Supplementary Tables

Table S1. Antibody concentration or dilution ratio.

REAGENT/RESOURCE	SOURCE	IDENTIFIER	DILUTION RATIOS
Anti-Acetyl-P53 (Lys120)	Affinity	Cat# AF3745; RRID:AB_2847059	1:2000 (WB)
Anti-Arg-1	HUABIO	Cat# ET1605-8; RRID:AB_3069721	1:2000 (WB)
Anti-α-Tubulin	Proteintech	Cat# 11224-1-AP; RRID:AB_2210206	1:2000 (WB)
Anti-β-actin	Proteintech	Cat# 66009-1-Ig; RRID:AB_2687938	1:2000 (WB)
Anti-CD14	Proteintech	Cat# 17000-1-AP; RRID:AB_2074048	1:200 (IF)
Anti-Flag-Tag	Abmart	Cat# M20008; RRID:AB_2713960	1:2000 (WB) 1:100 (Co-IP) 1:600 (IF)
Anti-Flag-Tag	MBL	Cat# M185-3L; RRID:AB_11123930	2 μg/sample (ChIP)
Anti-F4/80	CST	Cat# 70076s; RRID:AB_2799771	1:200 (IF)
Anti-GFP	Proteintech	Cat# 50430-2-AP; RRID:AB_11042881	1:2000 (WB)
Anti-HA-Tag	Proteintech	Cat# 51064-2-AP; RRID:AB_11042321	1:2000 (WB)
Anti-His-Tag	Wanleibio	Cat# WL05561	1:2000 (WB)
Anti-Histone-H3	CST	Cat# 9715s; RRID:AB_331563	1:2000 (WB)
Anti-iNOS	HUABIO	Cat# ER1706-89; RRID:AB_3069098	1:2000 (WB)
Anti-Myc-Tag	Abbkine	Cat# ABT2060 ; RRID:N/A	1:2000 (WB) 5 μg/mL (Co-IP)

REAGENT/RESOURCE	SOURCE	IDENTIFIER	DILUTION RATIOS
Anti-PD-1	CST	Cat# 84651s; RRID:AB_2800041	1:2000 (WB) 1:200 (IF)
Anti-PD-L1	Bio X Cell	Cat# BE0101; RRID:AB_10949073	1:200 (IF)
Anti-PFKM	Proteintech	Cat# 55028-1-AP; RRID:AB_10858390	1:2000 (WB) 1:200 (IF)
Anti-p53	CST	Cat# 2524s ; RRID:AB_331743	1:2000 (WB) 1:200 (IF) 1:200 (ChIP)
HRP-conjugated Goat Anti-Mouse IgG (H+L)	Proteintech	Cat# SA00001-1; RRID:AB_2722565	1:5000 (WB) 2 µg/sample (ChIP)
HRP-conjugated Goat Anti-Rabbit IgG (H+L)	Proteintech	Cat# SA00001-2; RRID:AB_2722564	1:5000 (WB)
IPKine™ HRP, Goat Anti-Mouse IgG HCS	Abbkine	Cat# A25112; RRID:AB_2922983	1:5000 (WB)
IPKine™ HRP, Goat Anti-Mouse IgG LCS	Abbkine	Cat# A25012; RRID:AB_2737290	1:5000 (WB)
IPKine™ HRP, Goat Anti-Rabbit IgG HCS	Abbkine	Cat# A25222; RRID:AB_2922982	1:5000 (WB)
IPKine™ HRP, Mouse Anti-Rabbit IgG LCS	Abbkine	Cat# A25022; RRID:AB_2893334	1:5000 (WB)
APC anti-mouse F4/80 Antibody	Biolegend	Cat# 123115; RRID:AB_893493	1:200 (FACS)
PE/Cyanine7 anti-mouse Ly-6G Antibody	Biolegend	Cat# 127617; RRID:AB_1877262	1:200 (FACS)
488-Goat-Anti-Rabbit Recombinant Secondary Antibody	Proteintech	Cat# RGAR002; RRID:AB_3073506	1:200 (IF)

CST: Cell Signaling Technology

Table S2. Primer sequences for Real-time RT-PCR.

Gene name	Direction	Sequence (5'→3')
Actb	F	GTGGGAATGGGTCAGAAGGA
	R	CTTCTCCATGTCGTCCCAGT
Akt2	F	ACGTGGTGAATACATCAAGACC
	R	GCTACAGAGAAATTGTTCAGGGG
Birc3	F	ACGCAGCAATCGTGCATTTTG
	R	CCTATAACGAGGTCACTGACGG
Ctnnb1	F	ATGGAGCCGGACAGAAAAGC
	R	CTTGCCACTCAGGGAAGGA
Hk1	F	CCAAAATAGACGAGGCCGTA
	R	TTCAGCAGCTTGACCACATC
Hk2	F	GAAGATGATCAGCGGGATGT
	R	GCCAGTGGTAAGGAGCTCTG
Il6	F	CCGGAGAGGAGACTTCACAG
	R	TCCACGATTTCCCAGAGAAC
Itgam	F	CCATGACCTTCCAAGAGAATGC
	R	ACCGGCTTGTGCTGTAGTC
Ldha	F	TGTCTCCAGCAAAGACTACTGT
	R	GACTGTACTTGACAATGTTGGGA
Pak4	F	AGCAGAAGTTCACAGGGCTG
	R	CTGGATGGACGTGATGCAGG
Pdcd1	F	AGCAAGGACGACACTCTGAA
	R	GCTCTGGTGTCTTCTCTCGT
Pfkl	F	GGAGGCGAGAACATCAAGCC
	R	CGGCCTTCCCTCGTAGTGA
Pfkm	F	TGACACAGCACTGAACACCA
	R	AGCCACCCATAGTCTCGATG
Pfkp	F	GAAACATGAGGCGTTCTGTGT
	R	CCCGGCACATTGTTGGAGA
Pkm	F	CGCCTGGACATTGACTCTG
	R	GAAATTCAGCCGAGCCACATT
Pxn	F	CAAACGGCCAGTGTTCTTGTC
	R	TGTGTGGTTTCCAGTTGGGTA
Raf1	F	TGGACTCAAAGATGCGGTGTT
	R	AAAACCCGGATAGTATTGCTTGT
Tnf-α	F	ACGGCATGGATCTCAAAGAC
	R	GTGGGTGAGGAGCACGTAGT

Table S3. Primer Sequences for ChIP.

Gene name	Direction	Sequence (5'→3')
REA	F	CCTAGCCTTGACAGGGGTCT
	R	GCACTAGCTTGGTGGGGAAG
REB	F	CCTCAGTCCCTAGGAGGTGG
	R	CATCGTCTCGGGTCCTAGGA
REC	F	CATCGTCTCGGGTCCTAGGA
	R	ATTCCTGGTACCCCGATCGA
RED	F	GGACCCCATAGCAGGACAAG
	R	GGAGGGAGGAGACCTGTTCT

Table S4. Parameters related to nanobodies.

Sample	Hydrodynamic diameter	Protein molecular weight	Isoelectric point	Extinction coefficient
Nb07	0.2767 µm	14 kDa	8.58	1.987

Table S5. SPR Analysis of Nb07 binding to PFKM.

Association Rate Constant (ka)	Dissociation Rate Constant (kd)	Equilibrium Dissociation Constant (KD):	Maximum Binding Response (Rmax):	Association Time Constant (tc):
21,200 1/(M·s)	$4.9e^{-8}$ 1/s	$2.32e^{-12}$ M	343.6 RU	$4.62e^{8}$ s
